# Efficacy of digital interventions in social anxiety disorder: a systematic review and Bayesian network meta-analysis

**DOI:** 10.3389/fpsyt.2026.1883150

**Published:** 2026-07-10

**Authors:** Yang Li, Zeng-yun-ou Zhang, Li-ping He, Xiao-qiu Zhou, Bo Yu, Xue-min Huang, Dan Yang, Deng-mei Xia, Dan Wang

**Affiliations:** 1 Department of Psychiatry, Sichuan Mental Health Center (The Third Hospital of Mianyang), Mianyang, China; 2 Department of Dermatology, West China Second University Hospital, Sichuan University, Key Laboratory of Birth Defects and Related Diseases of Women and Children (Sichuan University), Ministry of Education, Chengdu, China

**Keywords:** Bayesian network meta-analysis, digital intervention, internet-based cognitive behavioral therapy, social anxiety disorder, systematic review

## Abstract

**Background:**

Social anxiety disorder (SAD) is characterized by a significant and persistent fear of social or performance situations. The prevalence of SAD has gradually increased recently, and the unique advantages of digital interventions (DIs) have gained traction in psychiatric disorders. However, there is currently no comprehensive review comparing the effectiveness of diverse DIs for SAD.

**Methods:**

Randomized controlled trials (RCTs) evaluating DIs for patients with SAD were identified by searching the PubMed, Cochrane Library, and Embase databases from January 1, 1995, to March 31, 2025. The study protocol for this network meta-analysis was registered in *PROSPERO*. Data were analyzed via Bayesian framework network meta-analysis.

**Results:**

Forty-two RCTs were included. The results showed that DIs exerted better efficacy than non-digital interventions and wait-list controls (WLC). Different forms of internet-based cognitive behavioral therapy (ICBT) demonstrated robust effects across all four outcomes. Internet-based cognitive therapy (ICT) yielded favorable effects in reducing social anxiety and depressive symptoms. VR showed relatively large effect sizes for improving quality of life.

**Conclusion:**

DIs can be recommended as adjunctive or combined treatments for SAD. Different forms of ICBT show consistent efficacy and can serve as the first-line option among digital interventions. We recommend promoting the application of DIs to expand treatment coverage for SAD and overcome the limitations of traditional psychotherapy.

**Systematic review registration:**

https://www.crd.york.ac.uk/PROSPERO/, identifier CRD420251077835.

## 1 Introduction

Social anxiety disorder (SAD), also termed as social phobia, involves intense fear, anxiety, and avoidance behaviors that exceed the actual threat posed by social or public situations (1, 2). The prevalence of SAD varies significantly across high-, middle-, and low-income countries (3–6). Some studies conducted in predominantly Islamic cultural settings report that the prevalence of SAD among the female population is approximately 30% (4). Treatment for SAD includes medication and psychotherapy (2). Among these options, medication often raises patient concerns due to potential adverse effects and addiction risk, while physical therapy can challenge patient compliance because of its lengthy duration and need for continuous intervention (7, 8).

Psychotherapy is typically recommended as the first-line treatment for mild to moderate SAD. However, it is often impeded by long waiting times for appointments and high treatment costs (9, 10). In this context, Digital Interventions (DIs) have emerged as a promising approach and have gradually become a research focus in psychiatry because of their unique advantages (11, 12). DIs refer to psychological treatments delivered through digital environments such as computers, the internet, smartphones, tablets, virtual reality, and wearable devices (12). These interventions integrate core techniques from therapeutic approaches like cognitive behavioral therapy (CBT) and psychodynamic therapy, designing and delivering tailored treatment plans based on patients’ heterogeneous characteristics (13). They offer advantages including enhanced accessibility, reduced stigma, immediate feedback, and increased self-efficacy, thereby addressing limitations of traditional psychological treatments (14). DIs are becoming a more acceptable and accessible option for patients with SAD (8, 15).

In the field of intervention studies for SAD, previous reviews have primarily concentrated on assessing the effectiveness of psychotherapy, pharmacotherapy, and combined interventions (16, 17). Reviews on DIs have mainly targeted internet-based cognitive behavioral therapy (ICBT), and few studies have yet evaluated the effectiveness of a broader range of DIs measures (18). Therefore, this study aims to conduct a comprehensive systematic review and network meta-analysis (NMA) of randomized controlled trials (RCTs) related to DIs. The objective is to assess the effectiveness of DIs in patients with SAD, address the existing research gap, and provide high-quality evidence to support clinical treatment.

## 2 Methods

### 2.1 Search strategy

Following the Preferred Reporting Items for Systematic Reviews and Meta-Analyses Protocols (PRISMA-P) guidelines, we searched the PubMed, Cochrane Library, and Embase databases for articles published from January 1, 1995, to March 31, 2025 (19, 20). To reduce retrieval bias, we additionally performed supplementary searches in PsycINFO and Web of Science, and screened relevant grey literature. No extra eligible RCTs were identified after the supplementary search. The study protocol for this NMA was registered in PROSPERO (CRD420251077835). Detailed search terms are shown in **Supplementary Table 1**. Inclusion criteria: (1) patients diagnosed with SAD; (2) RCTs; (3) the experimental group must include at least one DI (e.g., computer, mobile phone, software, VR, wearable device, etc.); (4) the control group includes another active intervention, a wait-list group, or a placebo group; (5) valid social anxiety symptoms scales; (6) English-Language publication. Exclusion Criteria: (1) SAD combined with other mental disorders; (2) studies not evaluating DI efficacy for SAD; (3) non-RCTs, (4) studies with unavailable data; (5) duplicate publications; (6) ongoing trials.

### 2.2 Data extraction

Two researchers independently conducted literature searches and used EndNote 2020 software to remove duplicate records. They independently screened titles and abstracts to identify articles requiring full-text review. They then independently assessed full texts for eligibility. Disagreements were resolved through discussion with a third investigator. The extracted information included the first author, year of publication, country income category, age, sample size, intervention, remission counts and mean and standard deviation of post-treatment scores for social anxiety symptoms, depressive symptoms, and quality of life.

### 2.3 Risk of bias assessment

Two researchers independently assessed the risk of bias using Review Manager 5.4 (ROB) in accordance with the Cochrane Handbook for Systematic Reviews of Interventions. The assessment covered randomized sequence generation, allocation concealment, blinding of participants and personnel, blinding of outcome assessment, incomplete outcome data, selective reporting, and other biases. Trials were classified as high-risk, low-risk, or uncertain. Disagreements were resolved with a third investigator.

### 2.4 Heterogeneity and inconsistency

We assessed heterogeneity using the Cochrane Q-test with 95% *CrI* for *I²*. A random-effects network meta-analysis model was adopted when substantial heterogeneity was detected. Global inconsistency was evaluated by comparing the Deviance Information Criterion (DIC) values between the inconsistency model and the consistency model. Model convergence was checked using trace plots, density plots, and the Brooks-Gelman-Rubin diagnostics. The transitivity assumption was evaluated by comparing key baseline characteristics (age, gender, baseline severity, country income level, comorbidity) across intervention groups to ensure comparability.

### 2.5 Statistical analysis

This study utilized R software to conduct network meta-analysis (*NMA*) within a Bayesian framework to evaluate the efficacy of different DIs. For continuous outcomes including social anxiety symptoms, depressive symptoms and quality of life, standardized mean differences (*SMDs*) with 95% credible intervals (*CrIs*) were calculated to pool effect sizes. When multiple assessment scales were used in a single study, the most commonly adopted scale across included trials was selected to unify outcome measures. Effect sizes were interpreted according to *Hedges’ g*, with small (0.2), medium (0.5) and large (0.8) effect thresholds. For binary outcomes (remission rate), odds ratios (*ORs*) with 95% *CrIs* were calculated.

The Surface Under the Cumulative Ranking Curve (SUCRA) was used to rank the efficacy of interventions, with values ranging from 0% to 100%; a higher SUCRA value indicated a better ranking.

We adopted a random-effects Bayesian network meta-analysis model with vague prior distributions. Three independent Markov Chain Monte Carlo (MCMC) chains were run. The burn-in period was set as 50,000 iterations, followed by a further 100,000 sampling iterations with a thinning interval of 10. Convergence was confirmed via trace plots, density plots and Brooks-Gelman-Rubin diagnostics. Node-splitting analysis was performed to evaluate local inconsistency between direct and indirect comparisons.

Data preprocessing was performed using Stata MP17, and the R 4.5.1 software with the gemtc package was used for all Bayesian network meta-analyses.

## 3 Results

### 3.1 General characteristics

**Figure 1:**
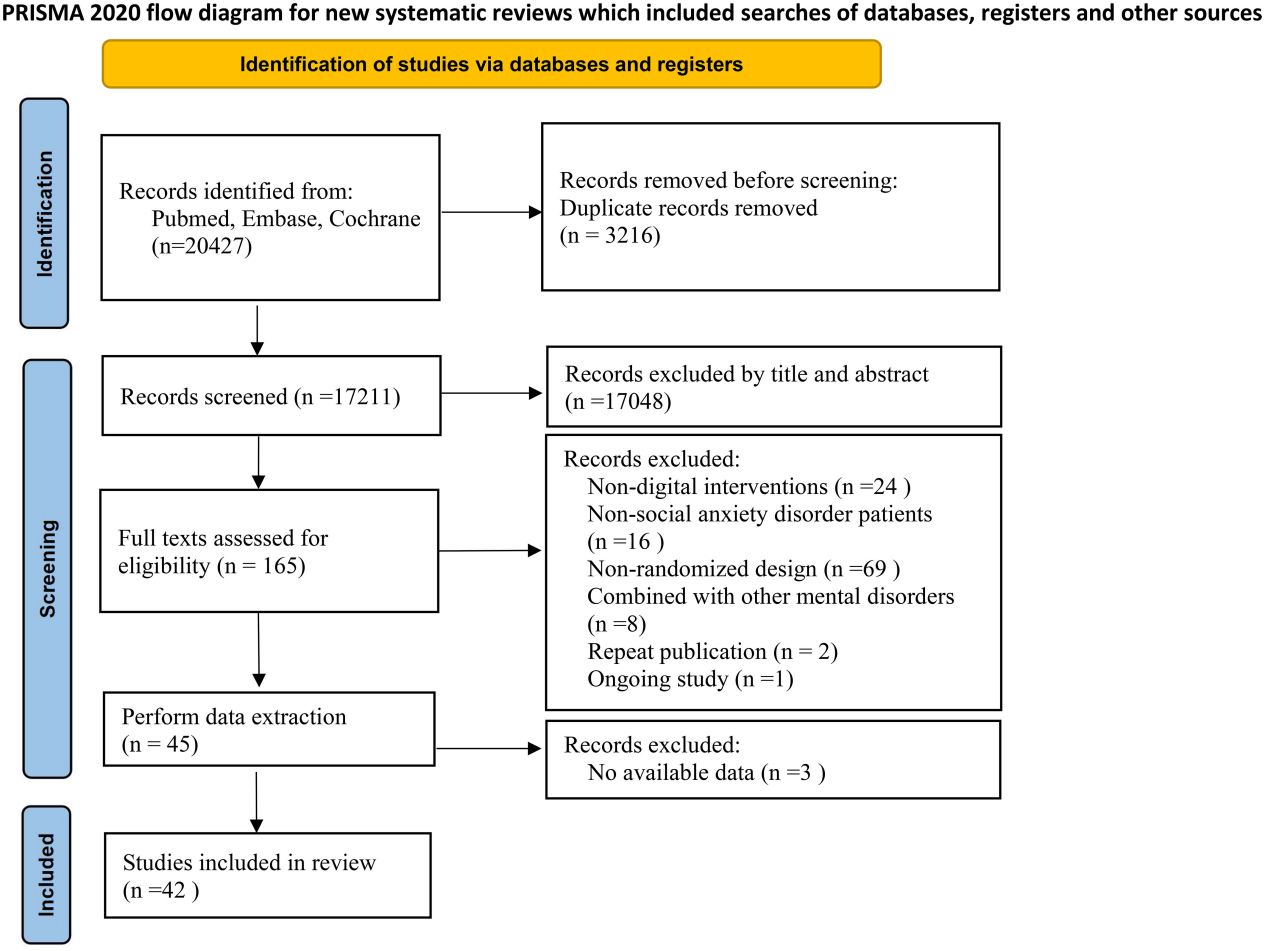
Literature search and selection.

We identified 20,427 publications; 42 RCTs were included (**Figure 1**) (21–62). The included studies were published between 2006 and 2025; mean age 6.2–43.6 years; sample size 19–204; total 3,696 SAD patients. 42 studies reported social anxiety scores, 27 depression, 14 quality of life, 23 remission.

Our NMA included 9 digital interventions (DIs), 5 non-digital interventions (non-DIs), and 1 wait-list control (WLC).

Nine digital interventions (DIs):

1. internet-based cognitive behavioral therapy (ICBT)
2. specific internet-based cognitive behavioral therapy (SICBT)
3. internet-based psychodynamic therapy (IPDT)
4. internet-based interpersonal psychotherapy (IIPT)
5. virtual reality exposure therapy (VR)
6. virtual reality attention guidance (VRAG)
7. internet coaching approach behavior and Leading by modeling (ICALM)
8. internet-based cognitive therapy (ICT)
9. internet-based supportive therapy(ISUPPROT)

Five non-digital interventions: face-to-face cognitive behavioral therapy (CBT), social effectiveness therapy (SET), exposure therapy (ET), cognitive bias modification (CBM), building closer friendships (BCF).

In addition, several less common interventions (MEMI, SBRC, AT) were identified in individual trials. Due to their extremely small sample sizes and limited research evidence, these interventions were merged into corresponding predefined categories in the network meta-analysis. All intervention abbreviations are uniformly defined in the figure legends and **Supplementary Materials**.

Number of RCTs per DI category:

ICBT: n=22 RCTs; SICBT: n=7; IPDT: n=1; IIPT: n=1; VR: n=8; VRAG: n=1; ICALM: n=1; ICT: n=3; ISUPPROT: n=1

Notably, several intervention nodes (IPDT, IIPT, VRAG, ICALM, ISUPPROT) were supported by only one RCT, and ICT was based on three RCTs. The unbalanced number of trials across nodes may lead to unstable SUCRA rankings, thus relevant ranking results should be interpreted with caution.

Studies originated from 15 countries classified by World Bank income criteria: high-income (n=13), middle-income (n=2) (**Supplementary Figure 1**). Detailed characteristics are in **Supplementary Table 2**. Most studies lacked long-term follow-up; such data were not included.

### 3.2 Primary and secondary outcomes

**Figure 2:**
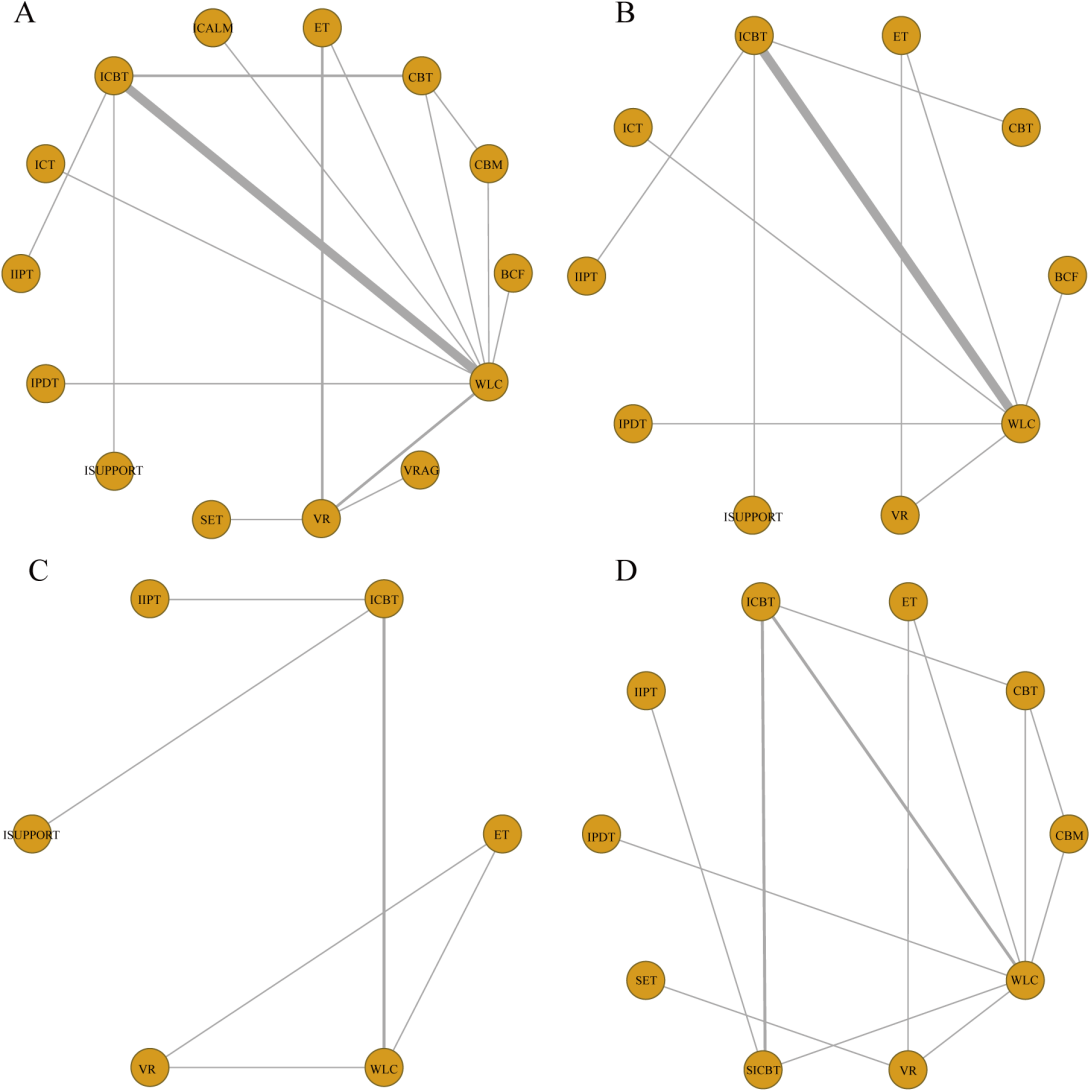
The network plots. **(a)** Social anxiety symptoms; **(b)** Depression symptoms; **(c)** Quality of Life; **(d)** Remission rates. BCF, Building closer friendships; CBM, Cognitive bias modification; CBT, Cognitive behavioral therapy; ET, Exposure therapy; ICBT, Internet-based cognitive behavioral therapy; ICT, Internet-based cognitive therapy (Clark and Wells model); ICALM, Internet coaching approach behavior and Leading by modeling; IIPT, Internet-based Interpersonal psychotherapy; IPDT, Internet-based psychodynamic therapy; SET, Social effectiveness therapy; SICBT, Specific internet-based cognitive behavioral treatment; VR, Virtual reality; VRAG, Virtual reality attention guidance; WLC, Wait-list controls.

**Figure 3:**
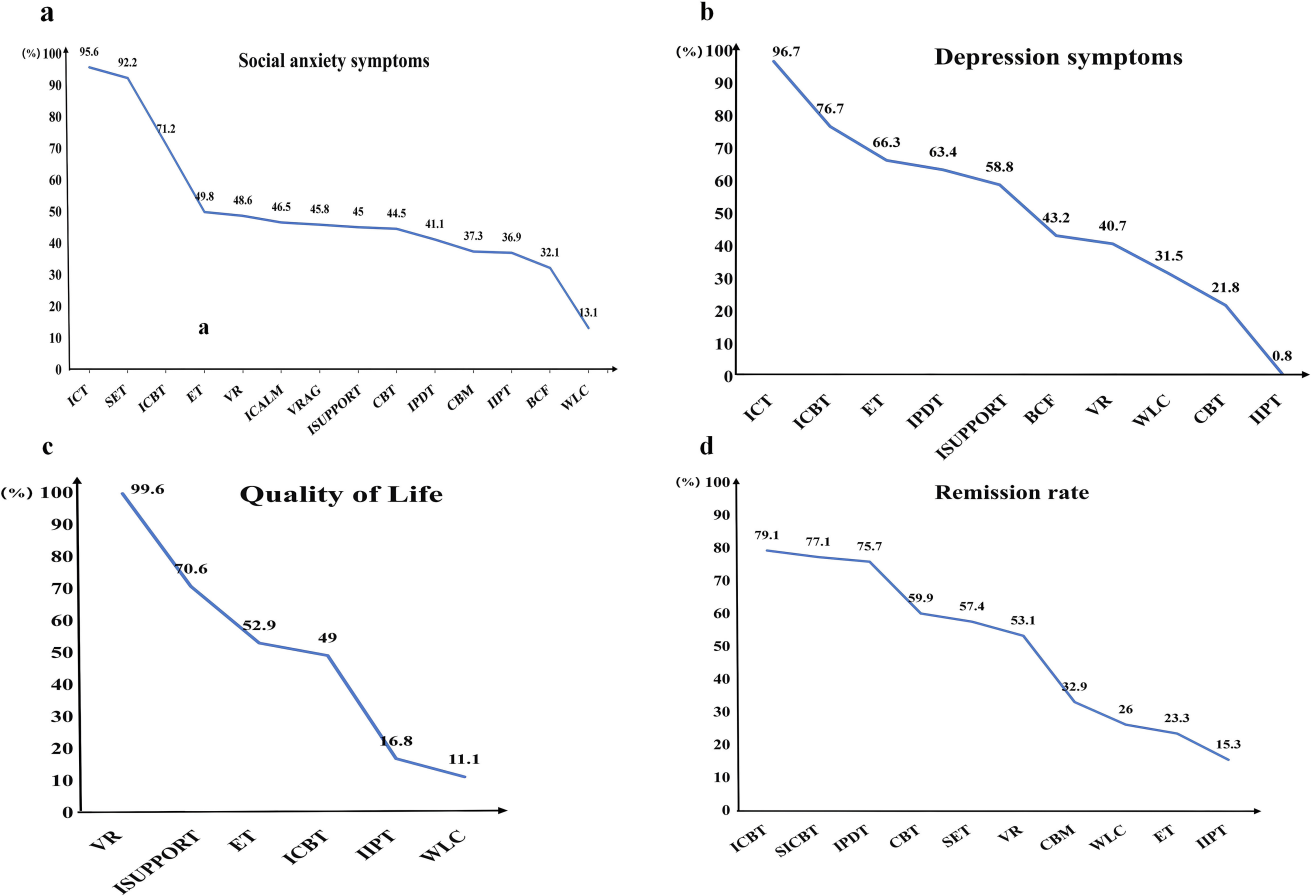
SUCRA ranking line charts. **(a)** Social anxiety symptoms; **(b)** Depression symptoms; **(c)** Quality of Life; **(d)** Remission rates.

**Figure 4:**
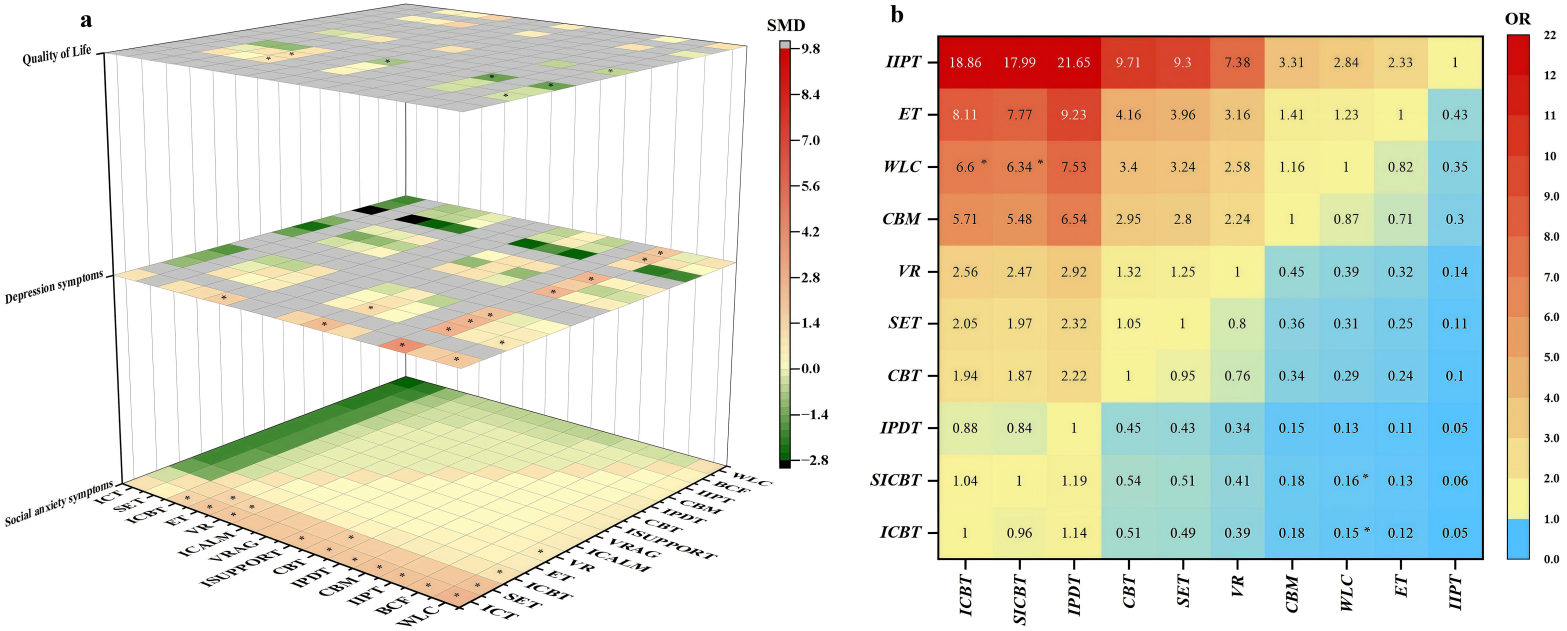
Heat maps. **(a)** Continuous variables. **(b)** Remission rates. *The intervention measures were compared pairwise and showed significant differences. The darker the color, the more significant the difference between the two.

**Figures 2**–**4** present network diagrams, SUCRA rankings, and heat maps for social anxiety, depressive symptoms, quality of life, and remission rate. DIs were more effective than non-DIs and WLC. Different forms of ICBT showed strong effectiveness; IIPT ranked lowest across four indicators.

In social anxiety NMA: ICT (*SMD*: −2.59, 95% *CrI* −4.05 to −1.14), SET (*SMD*: −2.27, −3.78 to −0.76), and ICBT (*SMD*: −1.03, −1.39 to −0.68) were superior to WLC (**Supplementary Figure 2**).

In depression NMA: ICT (*SMD*: −1.92, −3.27 to −0.59) and ICBT (*SMD*: −0.83, −1.25 to −0.48) were superior to WLC. ICT showed a numerically larger point estimate than ICBT for reducing social anxiety and depressive symptoms. However, the overlapping 95% *CrIs* indicated no statistically significant difference between the two interventions (**Supplementary Figure 3**).

We found no statistically significant differences between face-to-face CBT and WLC for both social anxiety symptoms (*SMD*: −0.57, 95% *CrI*: −0.27 to 0.08) and depressive symptoms (*SMD*: 0.43, −0.89 to 1.69). This finding was inconsistent with results from previous studies. The discrepancy may be attributed to the small number of relevant trials and high heterogeneity among included CBT studies, which was elaborated further in the Discussion section (**Supplementary Figures 2**, **3**).

In quality of life NMA: VR (*SMD*: 1.54, 0.83 to 2.25), ISUPPROT (*SMD*: 0.64, 0.18 to 1.10), and ICBT (*SMD*: 0.39, 0.21 to 0.58) were superior to WLC. Although VR presented the largest effect size for improving quality of life, this result should be regarded as preliminary. It was based on only 8 RCTs with a relatively wide credible interval, and further verification with larger samples is required (**Supplementary Figure 4**).

In remission NMA: ICBT (*OR*: 6.6, 95% *CrI* 2.45–20.33) and SICBT (*OR*: 6.34, 1.72–26.37) were superior to WLC (**Supplementary Figure 5**).

### 3.3 Risk of bias

**Figure 5:**
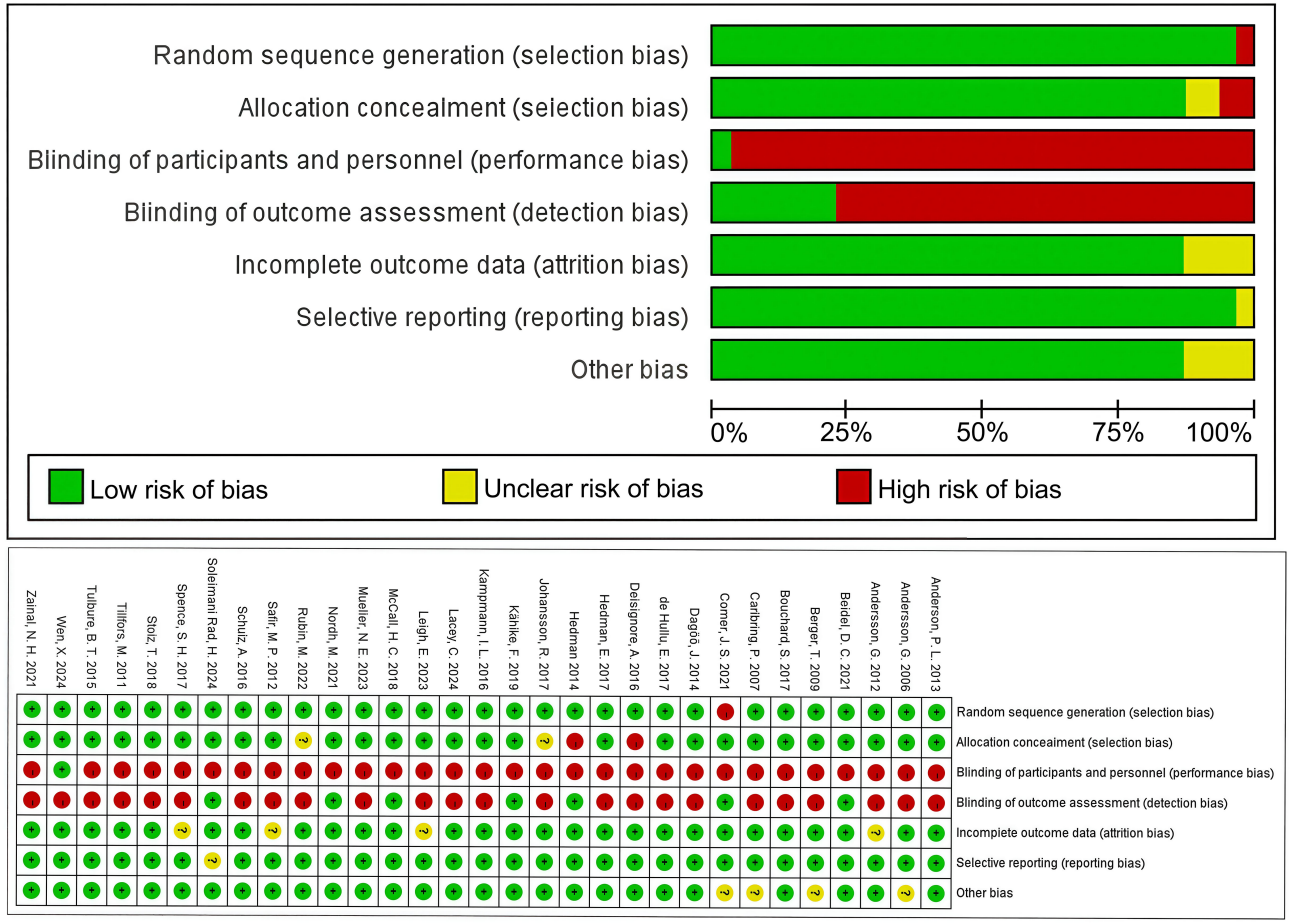
Risk of bias summary for efficacy outcome.

Most studies were at low risk for randomization but high risk for blinding due to intervention nature. Most used self-report scales, leading to unclear risk for outcome assessor blinding (**Figure 5**).

### 3.4 Inconsistency and sensitivity

After excluding high-risk studies, NMA showed good convergence (**Supplementary Figure 7**). For high heterogeneity, random-effects model was used (**Supplementary Figure 8**). Consistency and inconsistency models were tested; DIC difference < 5 indicated good consistency. Direct and indirect comparisons were not significantly different (all *P* > 0.05; **Supplementary Figure 9**).

We further performed a prespecified sensitivity analysis by excluding all studies recruiting participants younger than 18 years to assess the impact of age heterogeneity. A total of 9 trials focusing on children and adolescents were removed. After exclusion, the number of eligible studies decreased to 21 for social anxiety symptoms, 16 for depressive symptoms, 6 for quality of life, and 13 for remission rate, respectively.

All Bayesian models adopted four Markov Chain Monte Carlo chains, with 50,000 burn-in iterations and 20,000 sampling iterations. The maximum Potential Scale Reduction Factor (PSRF) across all outcomes was below 1.05, indicating satisfactory model convergence.

For most core interventions including ICBT and VR, effect sizes, 95% *CrIs* and SUCRA rankings were highly consistent between the primary analysis and sensitivity analysis. Several interventions (ICT, SET, ICALM, CBM, ISUPPORT) disappeared from the adult-only network, because these interventions were exclusively investigated in child and adolescent populations. Minor fluctuations in effect estimates and rankings of individual sparse nodes were attributed to the reduced sample size and sparse network structure after exclusion. Although the network for quality of life became relatively small, its core findings remained stable.

Overall, the main conclusions of this network meta-analysis were robust. Detailed results of the sensitivity analysis are presented in **Supplementary Tables 3**–**8**. The corresponding forest plot is shown in **Supplementary Figure 10**.

## 4 Discussion

In this Bayesian NMA of 42 RCTs (3,696 participants), 15 interventions were compared across four outcomes. DIs were generally more effective than non-DIs and WLC for improving social anxiety, depressive symptoms, quality of life, and remission. These findings indicated that DIs demonstrated significant effectiveness in all four indicators. They outperformed both non-DIs and WLC.

Notably, guided digital interventions (with therapist support, feedback, or supervision) generally yielded larger effect sizes than unguided (self-guided) digital interventions, which is consistent with existing clinical evidence (63). Therapist-guided delivery can improve treatment adherence, reduce dropout rates, and enhance symptom improvement compared with fully automated self-help programs (64, 65). This difference underscores the importance of human support in digital interventions for social anxiety disorder (66, 67).

Previous studies have established CBT as an effective treatment for SAD, and clinical guidelines recommend CBT as the first-line intervention (68, 69). Kampmann’s review found similar improvements in social anxiety and depression symptoms, which persisted at follow-up (70). A review of children and adolescents also indicated that ICBT was the most effective psychotherapy for SAD, consistent with our findings (71). Our research found that both ICT and ICBT significantly improve social anxiety and depressive symptoms, which is consistent with previous findings.

To clarify key interventions:

- CBT: conventional in-person, face-to-face psychotherapy delivered in clinical settings.
- ICBT: standard CBT protocols delivered via the internet, websites, or mobile applications.
- SICBT: structured, disorder-specific ICBT with standardized modules tailored explicitly for social anxiety disorder.
- ICT: a digital adaptation of Clark and Wells’ cognitive therapy model, which focuses specifically on cognitive mechanisms of social anxiety disorder rather than general CBT techniques.

ICT had a larger numerical effect size than ICBT, while no significant between-group difference was observed according to overlapping credible intervals. Cognitive therapy (CT), a specific type of CBT developed by Clark and Wells in 1995, is tailored for SAD (72). ICT, as a digital extension of CT, addresses limitations of traditional CBT regarding time, accessibility, and resource allocation (68). The results indicated no significant difference between ICT and ICBT when compared to SET, and existing evidence was insufficient to support SET’s superiority over other interventions. While SET has been identified as a specific intervention for SAD, previous studies have shown it to be effective for both adolescents and adults (34). SET was classified as an independent intervention because it combines CBT principles with social skills training and has distinct module structure, procedures, and therapeutic targets compared with standard CBT. It was not merged into CBT to maintain clinical homogeneity and consistency with existing classification systems in network meta-analyses (9).

The non-significant difference between conventional face-to-face CBT and WLC in our analysis may be explained by two reasons. First, only a small number of RCTs focused on traditional CBT were included, resulting in insufficient statistical power. Second, the included CBT trials were highly heterogeneous, including group therapy and individual therapy with different session durations and protocols, which may mask the true therapeutic effect. Our NMA compared remission across DIs for the first time. The results revealed significant differences between ICBT and SICBT compared to WLC. This indicates that various forms of ICBT not only improve social anxiety and depressive symptoms but also enhance the clinical remission rate of SAD (73, 74). Therefore, ICBT may serve as a supplementary approach to traditional CBT, and if SET can be digitized in the future, it may provide new options for interventions targeting SAD (75).

Relatively few prior studies have examined the relationship between VR and quality of life in SAD. This review suggested that VR may improve the quality of life for individuals with SAD. VR yielded a relatively large effect size for improving quality of life among all interventions. Nevertheless, given the limited number of relevant trials and wide credible intervals, this finding cannot be generalized definitively. This improvement may be attributed to VR’s ability to help patients confront their feared social situations through safe exposure, while repeated training addresses the challenges associated with social interaction, ultimately aiding patients in reintegrating into society and enhancing their overall quality of life (76, 77). The study also found that, compared with WLC, ICBT significantly improves quality of life. Kampmann’s review also indicated that ICBT had a moderate effect size in enhancing quality of life, consistent with our findings (70). However, the literature on quality of life in this context remains limited. Additionally, this study is exploratory in nature (78). Therefore, caution is warranted when generalizing these results. Future research should further investigate the quality of life in SAD through diverse interventions.

Several interventions (IPDT, IIPT, VRAG, ICALM, ISUPPROT) were only reported in a single RCT. Their SUCRA rankings are unstable and should be interpreted with great caution.

IIPT ranked lowest among the four indicators, likely due to a mismatch between the intervention mode of IIPT and the treatment needs of SAD. IIPT focuses on four core areas in interpersonal relationships: grief coping, dispute mediation, role transition and emotional sensitivity processing (79). Its intervention approach does not directly target the symptoms of SAD but aims to improve interpersonal functioning indirectly (80). This indirect intervention model, when applied to patients with SAD, may lead to a distraction from the primary treatment focus, increasing the complexity and cognitive load of the therapy (81). If patients fail to adhere to the treatment plan, achieving the desired outcomes will be difficult. Moreover, this may worsen social anxiety symptoms and lead to worse outcomes than those observed in the WLC group. Therefore, when selecting an intervention for SAD, it is crucial to carefully evaluate the suitability of the approach to avoid potential risks (82).

Reported dropout rates across included studies ranged from 8% to 22%, which was comparable to previous digital mental health research. Adherence rates were generally acceptable, though guided interventions showed higher adherence and lower dropout than unguided interventions. These data support the acceptability and feasibility of digital interventions in real-world clinical settings.

Digital interventions can serve as useful supplementary treatments for patients with mild to moderate SAD, especially for those who face barriers to in-person psychotherapy (83, 84). Appropriate online supervision should be integrated into digital programs to improve adherence and therapeutic effects (31).

## 5 Limitations

Several limitations of this study should be acknowledged. First, most included RCTs were non-double-blind and relied on self-report scales, which may introduce potential response bias. Second, although we initially searched three mainstream electronic databases, supplementary searches in PsycINFO, Web of Science and grey literature were conducted afterward to reduce retrieval bias. Third, the enrolled participants covered children, adolescents and adults across a wide age range. A number of included trials adopted mixed-age recruitment designs, and the number of studies focusing purely on specific age groups was limited. For these reasons, we did not perform formal subgroup analysis by age, so the pooled results should be interpreted cautiously when applied to different age populations. In addition, we conducted a sensitivity analysis by excluding juvenile-related trials, which verified the robustness of our primary results. However, partial outcome networks remained sparse after exclusion, and formal age-stratified subgroup analysis was not feasible due to limited single-age trials and mixed-age study designs.

Fourth, the number of trials varied greatly across different intervention nodes. Several interventions including IPDT, IIPT, VRAG, ICALM and ISUPPROT were only supported by a single RCT, while ICT was based on merely three trials. The unbalanced sample size across nodes led to unstable SUCRA rankings, and the relevant ranking results need to be interpreted with great caution. Fifth, not all trials were prospectively registered, which may increase the risk of selective reporting. Sixth, the transitivity assumption for network meta-analysis was verified by comparing baseline characteristics across groups, but unmeasured confounders could not be fully excluded.

Finally, intervention definitions for some digital interventions differed slightly across individual studies. In addition, limited long-term follow-up data were available in the included literature, so we could not evaluate the sustained therapeutic effects of these interventions.

## 6 Conclusion

Digital interventions (DIs) demonstrate superior efficacy compared with non-digital interventions and wait-list controls for treating social anxiety disorder (SAD), and can be applied as adjunctive treatments in clinical practice. Guided digital interventions present better therapeutic effects and treatment adherence than unguided programs. Various forms of internet-based cognitive behavioral therapy (ICBT) show consistent efficacy across multiple outcome indicators, and can serve as a primary option among digital interventions for SAD.

Considering the wide age range of participants and heterogeneous study designs in the included literature, the overall findings should be applied to different age groups with caution. Clinicians are advised to select appropriate interventions according to patients’ specific symptom profiles. Overall, digital interventions are feasible, accessible and effective tools to expand treatment coverage and compensate for the shortcomings of traditional psychological therapies for SAD.

## Data Availability

The original contributions presented in the study are included in the article/**Supplementary Material**. Further inquiries can be directed to the corresponding authors.

## Supplementary material

Supplementary Figure 1 Hot spot mapping. Two studies simultaneously included researchers from Germany, Switzerland, and Austria, and a total of 349 individuals were not included in the heat map. Map lines delineate study areas and do not necessarily depict accepted national boundaries.

Supplementary Figure 2 Comparison of different intervention measures in terms of social anxiety symptoms. Significant results are in red.

Supplementary Figure 3 Comparison of different intervention measures in terms of depression symptoms. Significant results are in red.

Supplementary Figure 4 Comparison of the effects of different intervention measures on quality of life. Significant results are in red.

Supplementary Figure 5 Comparison of the remission rates of different intervention measures. Significant results are in red.

Supplementary Figure 6 Forest plots. **(a)** Social anxiety symptoms; **(b)** Depression symptoms; **(c)** Quality of Life; **(d)** Remission rates.

Supplementary Figure 7 Convergence map. **(a)** Diagnostic chart for social anxiety symptoms. **(b)** Quality of life concentration density map. **(c)** Density plot of depressive symptoms convergence. **(d)** Convergence diagnostic plots and density plots for the remission rates.

Supplementary Figure 8 Heterogeneity map. **(a)** Social anxiety symptoms; **(b)** Depression symptoms; **(c)** Quality of Life; **(d)** Remission rates.

Supplementary Figure 9 Consistency charts. **(a)** Social anxiety symptoms; **(b)** Remission rates.

Supplementary Figure 10 Forest plot for social anxiety symptoms: primary analysis versus age-excluded sensitivity analysis.
