# Supplementary material for: Efficacy of digital interventions in social anxiety disorder: a systematic review and Bayesian network meta-analysis
Source: Front Psychiatry. 2026 Jul 10;17:1883150. doi: 10.3389/fpsyt.2026.1883150 (PMC13397225; doi:10.3389/fpsyt.2026.1883150)
Supplement: Supplementary Figure 1 — Hot spot mapping. Two studies simultaneously included researchers from Germany, Switzerland, and Austria, and a total of 349 individuals were not included in the heat map. Map lines delineate study areas and do not necessarily depict accepted national boundaries. [file DataSheet1.zip › Supplementary table1.docx]

**Supplementary Table 1. Full search strategy for each database**

**1 PubMed**

#1 Search "Phobia, Social"[Mesh]

#2 Search ( “social anxiety”[Title/Abstract] OR interaction anxiety[Title/Abstract] OR “social activities anxiety”[Title/Abstract] OR “social contact anxiety”[Title/Abstract] OR “society anxiety”[Title/Abstract] OR social distress[Title/Abstract] OR social behaviors[Title/Abstract] OR social anxiousness[Title/Abstract] OR “social anxiety disorder”[Title/Abstract] OR social anxiety disorders[Title/Abstract])

#3 #1 or #2

#4 Search(telecommunications[Title/Abstract] OR digital technology[Title/Abstract] OR computers, handheld[Title/Abstract] OR reminder systems[Title/Abstract] OR internet-based intervention[Title/Abstract] OR mobile applications[Title/Abstract] OR artificial intelligence[Title/Abstract] OR virtual reality[Title/Abstract] OR exergaming[Title/Abstract] OR wearable electronic devices[Title/Abstract] OR telemedicine[Title/Abstract] OR Telecommunications[Title/Abstract] OR telecommunication*[Title/Abstract] OR telecare[Title/Abstract] OR digital health[Title/Abstract] OR digital tool[Title/Abstract] OR digital care[Title/Abstract] OR e-health[Title/Abstract] OR ehealth[Title/Abstract] OR internet*[Title/Abstract] OR internet‐based[Title/Abstract] OR web‐based[Title/Abstract] OR m-health[Title/Abstract] OR mhealth[Title/Abstract] OR mobile phone[Title/Abstract] OR tablet[Title/Abstract] OR online case‐based learning[Title/Abstract] OR mobile app*[Title/Abstract] OR mobileapp*[Title/Abstract] OR mobile health[Title/Abstract] OR online health[Title/Abstract] OR smartphone*[Title/Abstract] OR electronic monitoring[Title/Abstract] OR reminder device*[Title/Abstract] OR reminder system*[Title/Abstract] OR helping hand[Title/Abstract] OR mobile phone*[Title/Abstract] OR internet website*[Title/Abstract] OR e-mail contact*[Title/Abstract] OR eHealth intervention*[Title/Abstract] OR monitoring device*[Title/Abstract] OR mobile application*[Title/Abstract] OR digital assistant*[Title/Abstract] OR IoT[Title/Abstract] OR chat-bot[Title/Abstract] OR chatbot[Title/Abstract] OR health website*[Title/Abstract] OR virtual reality modeling[Title/Abstract] OR visual reality[Title/Abstract] OR virtual reality technology[Title/Abstract] OR virtual realization[Title/Abstract] OR virtual[Title/Abstract] OR virtue reality[Title/Abstract] OR virtual environment[Title/Abstract] OR e-sports[Title/Abstract] OR wearable[Title/Abstract] OR smartwatch[Title/Abstract] OR smart watch[Title/Abstract])

#5 Search ("Electronic Health Records"[Mesh] OR "Internet-Based Intervention"[Mesh] OR "Digital Health"[Mesh] OR "Wearable Electronic Devices"[Mesh] OR "Virtual Reality"[Mesh] OR "Virtual Reality Exposure Therapy"[Mesh] OR "Exergaming"[Mesh] OR "Artificial Intelligence"[Mesh] OR "Mobile Applications"[Mesh] OR "Mobile Applications"[Mesh] OR "Reminder Systems"[Mesh] OR "Telerehabilitation"[Mesh] "Telemedicine"[Mesh] OR "Computers, Handheld"[Mesh] OR "Digital Technology"[Mesh] OR "Telecommunications"[Mesh])

#6 #4 or #5

#7 #3 AND #6 [Publication date from 1995/01/01 to 2025/03/31]

**2 Cochrane**

#1 'social anxiety' OR 'interaction anxiety' OR 'society anxiety' OR 'social distress' OR 'social behavior' OR 'social anxiousness' OR 'social anxiety disorder':ti OR 'social anxiety' OR 'interaction anxiety' OR 'society anxiety' OR 'social distress' OR 'social behavior' OR 'social anxiousness' OR 'social anxiety disorder':ab

#2 MeSH descriptor: [Social Phobia] explode all trees

#3 #1 or #2

#4 'telecommunication' OR 'digital technology' OR 'computer' OR 'reminder system' OR 'internet-based intervention' OR 'artificial intelligence' OR 'virtual reality' OR 'wearable electronic device' OR 'digital health' OR 'digital tool' OR 'digital care' OR 'e health' OR 'internet' OR ‘internet‐based’ OR ‘web‐based’ OR 'm health' OR ‘tablet’ OR 'mobile health' OR 'online health' OR 'smartphone' OR 'electronic monitoring' OR 'reminder device' OR 'helping hand' OR 'mobile phone' OR 'internet website' OR 'monitoring device' OR 'mobile application' OR 'digital assistant' OR 'health website' OR 'virtual reality modeling' OR 'visual reality' OR 'virtual reality technology' OR 'virtual realization' OR ‘virtual’ OR 'virtue reality' OR 'virtual environment' OR ‘wearable’ OR ‘smartwatch’ OR 'smart watch':ti OR 'telecommunication' OR 'digital technology' OR 'computer' OR 'reminder system' OR 'internet-based intervention' OR 'artificial intelligence' OR 'virtual reality' OR 'wearable electronic device' OR 'digital health' OR 'digital tool' OR 'digital care' OR 'e health' OR 'internet' OR ‘internet‐based’ OR ‘web‐based’ OR 'm health' OR ‘tablet’ OR 'mobile health' OR 'online health' OR 'smartphone' OR 'electronic monitoring' OR 'reminder device' OR 'helping hand' OR 'mobile phone' OR 'internet website' OR 'monitoring device' OR 'mobile application' OR 'digital assistant' OR 'health website' OR 'virtual reality modeling' OR 'visual reality' OR 'virtual reality technology' OR 'virtual realization' OR ‘virtual’ OR 'virtue reality' OR 'virtual environment' OR ‘wearable’ OR ‘smartwatch’ OR 'smart watch':ab

#5 MeSH descriptor: [Digital Technology] explode all trees

#6 MeSH descriptor: [Virtual Reality] explode all trees

#7 MeSH descriptor: [Digital health] explode all trees

#8 #4 or #5 or #6 or #7

#9 #3 and #8 [Publication date from 1995/01/01 to 2025/03/31]

**3 Embase**

#1 'social anxiety'/exp OR 'social anxiety disorder'/exp OR 'social phobia'/exp

#2 'social anxiety' OR 'interaction anxiety' OR 'society anxiety' OR 'social distress' OR 'social behavior' OR 'social anxiousness' OR 'social anxiety disorder':ab,ti

#3 #1 OR #2

#4 'digital technology'/exp OR 'computer'/exp OR 'mobile application'/exp OR 'virtual reality'/exp OR 'telemedicine'/exp OR 'digital health'/exp OR 'internet'/exp OR 'mobile phone'/exp OR 'online health' OR 'tablet'/exp OR 'ehealth'/exp

#5 'computer'/mj OR 'telemedicine'/mj OR 'internet'/mj OR 'phone' OR 'tablet'/mj OR 'ehealth'/mj

#6 'telecommunication':ab,ti OR 'digital technology':ab,ti OR 'computer':ab,ti OR 'reminder system':ab,ti OR 'internet-based intervention':ab,ti OR 'artificial intelligence':ab,ti OR 'virtual reality':ab,ti OR 'wearable electronic device':ab,ti OR 'digital health':ab,ti OR 'digital tool':ab,ti OR 'digital care':ab,ti OR 'e health':ab,ti OR 'internet':ab,ti OR internet‐based:ab,ti OR web‐based:ab,ti OR 'm health':ab,ti OR tablet:ab,ti OR 'mobile health':ab,ti OR 'online health':ab,ti OR 'smartphone':ab,ti OR 'electronic monitoring':ab,ti OR 'reminder device':ab,ti OR 'helping hand':ab,ti OR 'mobile phone':ab,ti OR 'internet website':ab,ti OR 'monitoring device':ab,ti OR 'mobile application':ab,ti OR 'digital assistant':ab,ti OR 'health website':ab,ti OR 'virtual reality modeling':ab,ti OR 'visual reality':ab,ti OR 'virtual reality technology':ab,ti OR 'virtual realization':ab,ti OR virtual:ab,ti OR 'virtue reality':ab,ti OR 'virtual environment':ab,ti OR wearable:ab,ti OR smartwatch:ab,ti OR 'smart watch':ab,ti

#7 #4 OR #5 OR #6

#8 #3 AND #7 [Publication year from 1995 to 2025]
