# Supplementary material for: Efficacy of digital interventions in social anxiety disorder: a systematic review and Bayesian network meta-analysis
Source: Front Psychiatry. 2026 Jul 10;17:1883150. doi: 10.3389/fpsyt.2026.1883150 (PMC13397225; doi:10.3389/fpsyt.2026.1883150)
Supplement: Supplementary Figure 1 — Hot spot mapping. Two studies simultaneously included researchers from Germany, Switzerland, and Austria, and a total of 349 individuals were not included in the heat map. Map lines delineate study areas and do not necessarily depict accepted national boundaries. [file DataSheet1.zip › Supplementary table2.docx]

**Supplementary Table 2. Characteristics of studies used for analysis.**

| **Study** | **Year** | **Country** | **Age** | **Mean±SD** | **Treatment times** | **follow-up times** | **Sample size** | **Interventions** | **N** | **Controls** | **N** | **Outcomes** |
| --- | --- | --- | --- | --- | --- | --- | --- | --- | --- | --- | --- | --- |
| Rubin, M.[1] | 2025 | United States | NA | 19.15±1.5(y^#^) | 2w^$^ | 2w | 53 | iExposure AG | 13 | iExposure | 18 | A,B |
|  |  |  |  |  |  |  |  | iExposure ACT | 22 |  |  |  |
| Lacey, C.[2] | 2024 | New Zealand | 18y-64y | 34.65±3.61(y) | 6w | 18w | 120 | VR | 60 | WLC | 60 | A,B |
| Wen, X.[3] | 2024 | China | ＞18y | 27.8±6.82(y) | 8w | NA | 201 | SICBT | 78 | WLC | 43 | A,B |
|  |  |  |  |  |  |  |  | ICBT | 80 |  |  |  |
| Soleimani Rad, H.[4] | 2024 | Iran | 15y-19y | 17±1.08(y) | 14w | 3m^&^ | 54 | ICBT | 18 | WLC | 18 | A,B |
|  |  |  |  |  |  |  |  | CBT | 18 |  |  |  |
| Zainal, N. H.[5] | 2024 | Singapore | ＞18y | 21.84±3.37(y) | 2w | 1m | 191 | MEMI | 96 | SM | 95 | A,B |
| Schwob, J. T.[6] | 2023 | United States | NA | 19.39±0.64(y) | 1w | 1m | 82 | ET | 39 | SM | 43 | A |
| Biagianti, B.[7] | 2023 | United States | 14y-18y | NA | 12w | NA | 22 | SICBT | 15 | ICBT | 7 | A |
| Schittenhelm, J. M.[8] | 2023 | Germany | 18y-65y | NA | NA | NA | 165 | ICT | 61 | WLC | 43 | A,B,C,D |
|  |  |  |  |  |  |  |  | CT | 61 |  |  |  |
| Mueller, N. E.[9] | 2023 | United States | NA | 19.36±2.09(y) | 4w | 1m | 55 | BCF | 28 | WLC | 27 | A,B |
| Leigh, E.[10] | 2023 | United Kingdom | 14y-18y | 16.23±1.07(y) | 14w | 6m | 43 | ICT | 22 | WLC | 21 | A,B |
| Clark, D. M.[11] | 2023 | United Kingdom | 18y-65y | NA | 14w | 12m | 102 | ICT | 34 | WLC | 34 | A |
|  |  |  |  |  |  |  |  | CT | 34 |  |  |  |
| Rubin, M.[12] | 2022 | United States | 18y-65y | 22.71±11.46(y) | 2w | 3w | 21 | VRAG | 11 | WLC | 10 | A |
| Zainal, N. H.[13] | 2021 | United States | ＞18y | 23.30±9.32(y) | NA | 6m | 44 | VR | 26 |  | 18 | A |
| Beidel, D. C.[14] | 2021 | United States | 7y-12y | 9.55±1.84(y) | NA | NA | 42 | VR | 26 | SET | 16 | A,D |
| Comer, J. S.[15] | 2021 | United States | 3y-8y | 6.2±1.8(y) | 16w | 6m | 40 | ICALM | 20 | WLC | 20 | A,D |
| Nordh, M.[16] | 2021 | Sweden | 10y-17y | 14.05±2.09(y) | 10w | 3m | 103 | ICBT | 51 | ISUPPORT | 52 | A,B,C |
| Kim, M. K.[17] | 2022 | Korea | 19y-30y | 23.55±2.49(y) | 2w | NA | 52 | VR | 24 | WLC | 28 | A |
| Cougle, J. R.[18] | 2020 | United States | 18y-65y | 32.99±11.53(y) | 1m | 2m | 94 | SBRC | 48 | PCC | 46 | A |
| Kählke, F.[19] | 2019 | Switzerland,  Germany,Austria | ＞18y | 26.70±6.34(y) | 10w | 6m | 200 | ICBT | 100 | WLC | 100 | A,B,C |
| Stolz, T.[20] | 2018 | Germany | ＞18y | 34.76±11.41(y) | NA | NA | 150 | ICBT | 30 | WLC | 60 | A,B,D |
|  |  |  |  |  |  |  |  | ICBT | 60 |  |  |  |
| McCall, H. C.[21] | 2018 | Canada | 17y-46y | 21.86±5.51(y) | 4m | 4m | 101 | ICBT | 51 | WLC | 50 | A,C |
| Johansson, R.[22] | 2017 | Sweden | 20y-72y | 42.9±13.3(y) | 10w | 24m | 72 | IPDT | 36 | WLC | 36 | A,B,D |
| Gershkovich, M.[23] | 2017 | United States | 18y-55y | 31.5±9.95(y) | NA | NA | 42 | ICBT | 20 | ICBT | 22 | A,D |
| de Hullu, E.[24] | 2017 | Netherlands | 12y-16y | 14.1±0.65(y) | 2w | 24m | 200 | CBM | 73 | WLC | 58 | A,D |
|  |  |  |  |  |  |  |  | CBT | 69 |  |  |  |
| Spence, S. H.[25] | 2017 | Australia | 8y-17y | 11.29±2.67(y) | 12w | 6m | 125 | SICBT | 47 | WLC | 30 | A,D |
|  |  |  |  |  |  |  |  | ICBT | 48 |  |  |  |
| Bouchard, S.[26] | 2017 | Canada | 18y-65y | 34.5±11.9(y) | 12w | 6m | 39 | VR | 17 | WLC | 20 | A,B,D |
|  |  |  |  |  |  |  |  | ET | 22 |  |  |  |
| Delsignore, A.[27] | 2016 | Switzerland | NA | 35±0.6(y) | 15w | 12m | 91 | ICBT | 44 | CBT | 47 | A,B |
| Schulz, A.[28] | 2016 | Switzerland,  Germany,Austria | 18y-76y | 35.38±11.16(y) | 12w | 6m | 149 | SICBT | 60 | WLC | 29 | A,B,D |
|  |  |  |  |  |  |  |  | ICBT | 60 |  |  |  |
| Gingnell, M.[29] | 2016 | Sweden | NA | 33.2±8.8(y) | 9w | 15m | 48 | EICBT | 24 | PICBT | 24 | A,B,C |
| Kampmann, I. L.[30] | 2016 | Netherlands | 18y-65y | 36.89±11.59(y) | NA | 3m | 60 | VR | 20 | WLC | 20 | A,B,C,D |
|  |  |  |  |  |  |  |  | ET | 20 |  |  |  |
| Tulbure, B. T.[31] | 2015 | Romania | 18y-54y | 28.81±8.04(y) | 9w | 6m | 76 | ICBT | 38 | WLC | 38 | A,B,D |
| Hedman, E.[32] | 2014 | Sweden | NA | 35.35±11.3(y) | 15w | 48m | 126 | ICBT | 64 | CBT | 62 | A,D |
| Dagöö, J.[33] | 2014 | Sweden | 20y-65y | 36.81±11.4(y) | 9w | 3m | 52 | ICBT | 27 | IIPT | 25 | A,B,C,D |
| Boettcher, J.[34] | 2014 | Germany | ＞18y | 33.4±10.39(y) | 11w | NA | 133 | AT ICBT | 66 | CICBT | 67 | A,B,C,D |
| Safir, M. P.[35] | 2012 | Israel | NA | 27(y) | 12w | 12m | 88 | VR | 28 | WLC | 30 | A |
|  |  |  |  |  |  |  |  | ET | 30 |  |  |  |
| Anderson, P. L.[36] | 2013 | United States | 19y-69y | 39.03±11.26(y) | 8w | 12m | 97 | VR | 30 | WLC | 28 | A |
|  |  |  |  |  |  |  |  | ET | 39 |  |  |  |
| Neubauer, K.[37] | 2013 | Germany | 18y-65y | 39.56±7.88(y) | NA | 4m | 56 | AT | 28 | PP | 28 | A,B,D |
| Carlbring, P.[38] | 2012 | Sweden | ＞18y | 36.5±12.7(y) | 4w | 4m | 79 | AM | 40 | PP | 39 | A,C,D |
| Andersson, G.[39] | 2012 | Sweden | ＞18y | 38.25±11.08(y) | 9w | 12m | 204 | ICBT | 102 | WLC | 102 | A,B,C,D |
| Tillfors, M.[40] | 2011 | Sweden | 15y-21y | 16.5±1.6(y) | 9w | 12m | 19 | ICBT | 10 | WLC | 9 | A,B,C,D |
| Titov, N.[41] | 2010 | Sweden | NA | 43.6±14.6(y) | 11w | 3m | 113 | SICBT | 57 | ICBT | 56 | A,B,D |
| Berger, T.[42] | 2009 | Switzerland | 19y-43y | 28.9±5.3(y) | 10w | NA | 52 | ICBT | 31 | WLC | 21 | A,B,D |
| Tillfors, M.[43] | 2008 | Sweden | 19y-51y | 32.03±8.59(y) | 9w | 12m | 38 | SICBT | 19 | ICBT | 19 | A,B,C,D |
| Carlbring, P.[44] | 2007 | Sweden | 19y-52y | 32.65±9.07(y) | 9w | 12m | 57 | ICBT | 29 | WLC | 28 | A,B,C |
| Andersson, G.[45] | 2006 | Sweden | 18y-67y | 37.3±10.2(y) | 9w | 12m | 64 | SICBT | 32 | WLC | 32 | A,C,D |

N, Number; NA, Not Available; AG, Attention guidance; ACT, Attention control training; VR, virtual reality ; SICBT, Specific internet-based cognitive behavioral therapy; CBT, Cognitive behavioral therapy; MEMI, Mindfulness ecological momentary interventions; ET, Exposure therapy; ICT, Internet-based cognitive therapy(Clark and Wells model); CT, Cognitive therapy(Clark and Wells model); BCF, Building closer friendships; VRAG, Virtual reality attention guidance; ICALM, Internet coaching approach behavior and Leading by modeling; SBRC, Safety behavior reduction condition; ICBT, Internet-based cognitive behavioral therapy; IPDT, Internet-based psychodynamic therapy; CBM, Cognitive bias modification; EICBT, escitalopram and internet-based cognitive behavioral therapy; AT, Attention training; AM, Attention modification; WLC, Wait-list controls; SM, Self-monitoring; SET, Social effectiveness therapy for children; PCC, Present centered condition; PICBT, Placebo and internet-based cognitive behavioral therapy;IIPT, Internet-based Interpersonal psychotherapy; CICBT, Control training internet-based cognitive behavioral therapy; PP, Placebo probe; A, Social anxiety symptoms; B, Depression symptoms; C, Quality of Life; D, Remission rate; “^#^” means years;“^$^” means weeks; “^&^” means months.

**References**

1 Rubin M, Gebhardt E, Malloy L, Telch MJ. An initial test of a new self-guided internet-based intervention for social anxiety: iExposure. Cogn Behav Ther. 2025;54(1):1-16.

2 Lacey C, Frampton C, Beaglehole B. A self-guided virtual reality solution for social anxiety: Results from a randomized controlled study. J Psychiatr Res. 2024;180:333-39.

3 Wen X, Gou M, Chen H, Kishimoto T, Qian M, Margraf J, et al. The Efficacy of Web-Based Cognitive Behavioral Therapy With a Shame-Specific Intervention for Social Anxiety Disorder: Randomized Controlled Trial. JMIR Ment Health. 2024;11:e50535.

4 Soleimani Rad H, Goodarzi H, Bahrami L, Abolghasemi A. Internet-Based Versus Face-to-Face Cognitive-Behavioral Therapy for Social Anxiety Disorder: A Randomized Control Trial. Behav Ther. 2024;55(3):528-42.

5 Zainal NH, Tan HH, Hong RY, Newman MG. Is a brief mindfulness ecological momentary intervention more efficacious than a self-monitoring app for social anxiety disorder? A randomized controlled trial. J Anxiety Disord. 2024;104:102858.

6 Schwob JT, Newman MG. Brief imaginal exposure exercises for social anxiety disorder: A randomized controlled trial of a self-help momentary intervention app. J Anxiety Disord. 2023;98:102749.

7 Biagianti B, Conelea C, Dabit S, Ross D, Beard KL, Harris E, et al. A Mobile Application Adjunct to Augment Cognitive-Behavioral Group Therapy for Adolescents with Social Anxiety: Feasibility and Acceptability Results from the Wiring Adolescents with Social Anxiety via Behavioral Interventions Pilot Trial. J Child Adolesc Psychopharmacol. 2023;33(6):212-24.

8 Schittenhelm JM, von Borell C, Clément C, Schüller J, Stangier U, Hoyer J. Evaluation of a smartphone application for self-help for patients with social anxiety disorder: a randomized controlled study-SMASH. Trials. 2023;24(1):154.

9 Mueller NE, Cougle JR. Building Closer Friendships in social anxiety disorder: A randomized control trial of an internet-based intervention. J Behav Ther Exp Psychiatry. 2023;78:101799.

10 Leigh E, Clark DM. Internet-delivered therapist-assisted cognitive therapy for adolescent social anxiety disorder (OSCA): a randomised controlled trial addressing preliminary efficacy and mechanisms of action. J Child Psychol Psychiatry. 2023;64(1):145-55.

11 Clark DM, Wild J, Warnock-Parkes E, Stott R, Grey N, Thew G, et al. More than doubling the clinical benefit of each hour of therapist time: a randomised controlled trial of internet cognitive therapy for social anxiety disorder. Psychol Med. 2023;53(11):5022-32.

12 Rubin M, Muller K, Hayhoe MM, Telch MJ. Attention guidance augmentation of virtual reality exposure therapy for social anxiety disorder: a pilot randomized controlled trial. Cogn Behav Ther. 2022;51(5):371-87.

13 Zainal NH, Chan WW, Saxena AP, Taylor CB, Newman MG. Pilot randomized trial of self-guided virtual reality exposure therapy for social anxiety disorder. Behav Res Ther. 2021;147:103984.

14 Beidel DC, Tuerk PW, Spitalnick J, Bowers CA, Morrison K. Treating Childhood Social Anxiety Disorder With Virtual Environments and Serious Games: A Randomized Trial. Behav Ther. 2021;52(6):1351-63.

15 Comer JS, Furr JM, Del Busto C, Silva K, Hong N, Poznanski B, et al. Therapist-Led, Internet-Delivered Treatment for Early Child Social Anxiety: A Waitlist-Controlled Evaluation of the iCALM Telehealth Program. Behav Ther. 2021;52(5):1171-87.

16 Nordh M, Wahlund T, Jolstedt M, Sahlin H, Bjureberg J, Ahlen J, et al. Therapist-Guided Internet-Delivered Cognitive Behavioral Therapy vs Internet-Delivered Supportive Therapy for Children and Adolescents With Social Anxiety Disorder: A Randomized Clinical Trial. JAMA Psychiatry. 2021;78(7):705-13.

17 Kim MK, Eom H, Kwon JH, Kyeong S, Kim JJ. Neural effects of a short-term virtual reality self-training program to reduce social anxiety. Psychol Med. 2022;52(7):1296-305.

18 Cougle JR, Mueller NE, McDermott KA, Wilver NL, Carlton CN, Okey SA. Text message safety behavior reduction for social anxiety: A randomized controlled trial. J Consult Clin Psychol. 2020;88(5):445-54.

19 Kählke F, Berger T, Schulz A, Baumeister H, Berking M, Cuijpers P, et al. Efficacy and cost-effectiveness of an unguided, internet-based self-help intervention for social anxiety disorder in university students: protocol of a randomized controlled trial. BMC Psychiatry. 2019;19(1):197.

20 Stolz T, Schulz A, Krieger T, Vincent A, Urech A, Moser C, et al. A mobile app for social anxiety disorder: A three-arm randomized controlled trial comparing mobile and PC-based guided self-help interventions. J Consult Clin Psychol. 2018;86(6):493-504.

21 McCall HC, Richardson CG, Helgadottir FD, Chen FS. Evaluating a Web-Based Social Anxiety Intervention Among University Students: Randomized Controlled Trial. J Med Internet Res. 2018;20(3):e91.

22 Johansson R, Hesslow T, Ljótsson B, Jansson A, Jonsson L, Färdig S, et al. Internet-based affect-focused psychodynamic therapy for social anxiety disorder: A randomized controlled trial with 2-year follow-up. Psychotherapy (Chic). 2017;54(4):351-60.

23 Gershkovich M, Herbert JD, Forman EM, Schumacher LM, Fischer LE. Internet-Delivered Acceptance-Based Cognitive-Behavioral Intervention for Social Anxiety Disorder With and Without Therapist Support: A Randomized Trial. Behav Modif. 2017;41(5):583-608.

24 de Hullu E, Sportel BE, Nauta MH, de Jong PJ. Cognitive bias modification and CBT as early interventions for adolescent social and test anxiety: Two-year follow-up of a randomized controlled trial. J Behav Ther Exp Psychiatry. 2017;55:81-89.

25 Spence SH, Donovan CL, March S, Kenardy JA, Hearn CS. Generic versus disorder specific cognitive behavior therapy for social anxiety disorder in youth: A randomized controlled trial using internet delivery. Behav Res Ther. 2017;90:41-57.

26 Bouchard S, Dumoulin S, Robillard G, Guitard T, Klinger É, Forget H, et al. Virtual reality compared with in vivo exposure in the treatment of social anxiety disorder: a three-arm randomised controlled trial. Br J Psychiatry. 2017;210(4):276-83.

27 Delsignore A, Rufer M, Emmerich J, Weidt S, Brühl AB, Moergeli H. E-mail support as an adjunct to cognitive-behavioral group therapy for social anxiety disorder: Impact on dropout and outcome. Psychiatry Res. 2016;244:151-8.

28 Schulz A, Stolz T, Berger T. Internet-based individually versus group guided self-help treatment for social anxiety disorder: protocol of a randomized controlled trial. BMC Psychiatry. 2014;14:115.

29 Gingnell M, Frick A, Engman J, Alaie I, Björkstrand J, Faria V, et al. Combining escitalopram and cognitive-behavioural therapy for social anxiety disorder: randomised controlled fMRI trial. Br J Psychiatry. 2016;209(3):229-35.

30 Kampmann IL, Emmelkamp PM, Hartanto D, Brinkman WP, Zijlstra BJ, Morina N. Exposure to virtual social interactions in the treatment of social anxiety disorder: A randomized controlled trial. Behav Res Ther. 2016;77:147-56.

31 Tulbure BT, Szentagotai A, David O, Ștefan S, Månsson KN, David D, et al. Internet-delivered cognitive-behavioral therapy for social anxiety disorder in Romania: a randomized controlled trial. PLoS One. 2015;10(5):e0123997.

32 Hedman E, El Alaoui S, Lindefors N, Andersson E, Rück C, Ghaderi A, et al. Clinical effectiveness and cost-effectiveness of Internet- vs. group-based cognitive behavior therapy for social anxiety disorder: 4-year follow-up of a randomized trial. Behav Res Ther. 2014;59:20-9.

33 Dagöö J, Asplund RP, Bsenko HA, Hjerling S, Holmberg A, Westh S, et al. Cognitive behavior therapy versus interpersonal psychotherapy for social anxiety disorder delivered via smartphone and computer: a randomized controlled trial. J Anxiety Disord. 2014;28(4):410-7.

34 Boettcher J, Hasselrot J, Sund E, Andersson G, Carlbring P. Combining attention training with internet-based cognitive-behavioural self-help for social anxiety: a randomised controlled trial. Cogn Behav Ther. 2014;43(1):34-48.

35 Safir MP, Wallach HS, Bar-Zvi M. Virtual reality cognitive-behavior therapy for public speaking anxiety: one-year follow-up. Behav Modif. 2012;36(2):235-46.

36 Anderson PL, Price M, Edwards SM, Obasaju MA, Schmertz SK, Zimand E, et al. Virtual reality exposure therapy for social anxiety disorder: a randomized controlled trial. J Consult Clin Psychol. 2013;81(5):751-60.

37 Neubauer K, von Auer M, Murray E, Petermann F, Helbig-Lang S, Gerlach AL. Internet-delivered attention modification training as a treatment for social phobia: a randomized controlled trial. Behav Res Ther. 2013;51(2):87-97.

38 Carlbring P, Apelstrand M, Sehlin H, Amir N, Rousseau A, Hofmann SG, et al. Internet-delivered attention bias modification training in individuals with social anxiety disorder--a double blind randomized controlled trial. BMC Psychiatry. 2012;12:66.

39 Andersson G, Carlbring P, Furmark T. Therapist experience and knowledge acquisition in internet-delivered CBT for social anxiety disorder: a randomized controlled trial. PLoS One. 2012;7(5):e37411.

40 Tillfors M, Andersson G, Ekselius L, Furmark T, Lewenhaupt S, Karlsson A, et al. A randomized trial of Internet-delivered treatment for social anxiety disorder in high school students. Cogn Behav Ther. 2011;40(2):147-57.

41 Titov N, Andrews G, Schwencke G, Robinson E, Peters L, Spence J. Randomized controlled trial of Internet cognitive behavioural treatment for social phobia with and without motivational enhancement strategies. Aust N Z J Psychiatry. 2010;44(10):938-45.

42 Berger T, Hohl E, Caspar F. Internet-based treatment for social phobia: a randomized controlled trial. J Clin Psychol. 2009;65(10):1021-35.

43 Tillfors M, Carlbring P, Furmark T, Lewenhaupt S, Spak M, Eriksson A, et al. Treating university students with social phobia and public speaking fears: Internet delivered self-help with or without live group exposure sessions. Depress Anxiety. 2008;25(8):708-17.

44 Carlbring P, Gunnarsdóttir M, Hedensjö L, Andersson G, Ekselius L, Furmark T. Treatment of social phobia: randomised trial of internet-delivered cognitive-behavioural therapy with telephone support. Br J Psychiatry. 2007;190:123-8.

45 Andersson G, Carlbring P, Holmström A, Sparthan E, Furmark T, Nilsson-Ihrfelt E, et al. Internet-based self-help with therapist feedback and in vivo group exposure for social phobia: a randomized controlled trial. J Consult Clin Psychol. 2006;74(4):677-86.
