# Supplementary material for: Efficacy of digital interventions in social anxiety disorder: a systematic review and Bayesian network meta-analysis
Source: Front Psychiatry. 2026 Jul 10;17:1883150. doi: 10.3389/fpsyt.2026.1883150 (PMC13397225; doi:10.3389/fpsyt.2026.1883150)
Supplement: Supplementary Figure 1 — Hot spot mapping. Two studies simultaneously included researchers from Germany, Switzerland, and Austria, and a total of 349 individuals were not included in the heat map. Map lines delineate study areas and do not necessarily depict accepted national boundaries. [file DataSheet1.zip › Supplementary Tables3-8.docx]

**Supplementary Table 3. SAD severity: network estimates (SMD vs WLC), main vs age-excluded sensitivity analysis.**

| **Comparison (vs WLC)** | **Main analysis, SMD (95% CrI)** | **Age-excluded, SMD (95% CrI)** |
| --- | --- | --- |
| BCF | -0.29 (-1.61, 1.05) | -0.29 (-1.42, 0.83) |
| CBM | -0.41 (-1.55, 0.69) | — |
| CBT | -0.56 (-1.26, 0.07) | -0.06 (-0.87, 0.78) |
| ET | -0.67 (-1.35, 0.02) | -0.66 (-1.24, -0.08) |
| ICALM | -0.61 (-1.99, 0.76) | — |
| ICBT | -1.03 (-1.38, -0.68) | -0.90 (-1.25, -0.54) |
| ICT | -2.58 (-4.04, -1.12) | — |
| IIPT | -0.40 (-1.76, 0.95) | -0.27 (-1.42, 0.89) |
| IPDT | -0.49 (-1.77, 0.79) | -0.49 (-1.55, 0.57) |
| ISUPPORT | -0.58 (-1.90, 0.71) | — |
| SET | -2.28 (-3.76, -0.78) | — |
| VR | -0.65 (-1.23, -0.06) | -0.64 (-1.14, -0.15) |
| VRAG | -0.61 (-2.17, 0.96) | -0.60 (-1.97, 0.77) |
| sd.d | 0.57 (0.38, 0.87) | 0.45 (0.28, 0.74) |
| CrI, credible interval; SMD, standardized mean difference; OR, odds ratio; WLC, waiting-list control. Values are posterior median (95% CrI). | | |

SMD = standardized mean difference; OR = odds ratio; CrI = credible interval; WLC = wait-list control; τ = between-study standard deviation; SUCRA = surface under the cumulative ranking curve; PSRF = potential scale reduction factor. “—” indicates the treatment was not present in the age-excluded network (its supporting studies enrolled minors only).

**Supplementary Table 4. Depression: network estimates (SMD vs WLC), main vs age-excluded.**

| **Comparison (vs WLC)** | **Main analysis, SMD (95% CrI)** | **Age-excluded, SMD (95% CrI)** |
| --- | --- | --- |
| BCF | -0.16 (-1.42, 1.11) | -0.16 (-0.99, 0.66) |
| CBT | 0.43 (-0.90, 1.70) | 0.60 (-0.19, 1.39) |
| ET | -0.65 (-1.52, 0.22) | -0.62 (-1.19, -0.08) |
| ICBT | -0.83 (-1.25, -0.49) | -0.66 (-0.91, -0.43) |
| ICT | -1.92 (-3.28, -0.57) | — |
| IIPT | 2.10 (0.65, 3.49) | 2.27 (1.24, 3.25) |
| IPDT | -0.62 (-1.84, 0.60) | -0.62 (-1.34, 0.12) |
| ISUPPORT | -0.52 (-1.82, 0.70) | — |
| VR | -0.16 (-0.88, 0.57) | -0.17 (-0.62, 0.30) |
| sd.d | 0.50 (0.18, 0.96) | 0.21 (0.02, 0.56) |
| CrI, credible interval; SMD, standardized mean difference; OR, odds ratio; WLC, waiting-list control. Values are posterior median (95% CrI). | | |

**Supplementary Table 5. Quality of life: network estimates (SMD vs WLC), main vs age-excluded.**

| **Comparison (vs WLC)** | **Main analysis, SMD (95% CrI)** | **Age-excluded, SMD (95% CrI)** |
| --- | --- | --- |
| ET | 0.45 (-0.33, 1.25) | 0.46 (-0.52, 1.44) |
| ICBT | 0.39 (0.12, 0.67) | 0.44 (0.01, 0.88) |
| IIPT | 0.03 (-0.72, 0.79) | 0.07 (-0.94, 1.09) |
| ISUPPORT | 0.64 (-0.03, 1.31) | — |
| VR | 1.54 (0.70, 2.37) | 1.55 (0.52, 2.56) |
| sd.d | 0.13 (0.01, 0.57) | 0.18 (0.01, 1.06) |
| CrI, credible interval; SMD, standardized mean difference; OR, odds ratio; WLC, waiting-list control. Values are posterior median (95% CrI). | | |

**Supplementary Table 6. Response: network estimates (OR vs WLC), main vs age-excluded.**

| **Comparison (vs WLC)** | **Main analysis, OR (95% CrI)** | **Age-excluded, OR (95% CrI)** |
| --- | --- | --- |
| CBM | 1.16 (0.10, 14.15) | 1.15 (0.18, 7.30) |
| CBT | 3.40 (0.47, 24.17) | 3.49 (0.78, 14.86) |
| ET | 0.81 (0.09, 6.07) | 3.88 (0.42, 39.87) |
| ICBT | 6.65 (2.47, 20.64) | 5.91 (2.74, 15.65) |
| IIPT | 0.34 (0.01, 9.58) | 0.31 (0.02, 4.83) |
| IPDT | 7.47 (0.46, 124.69) | 7.54 (0.93, 63.92) |
| SET | 3.22 (0.10, 107.43) | — |
| SICBT | 6.36 (1.71, 26.73) | 5.49 (1.71, 21.70) |
| VR | 2.59 (0.35, 20.83) | 0.91 (0.10, 7.83) |
| sd.d | 3.05 (1.71, 8.46) | 1.99 (1.08, 5.76) |
| CrI, credible interval; SMD, standardized mean difference; OR, odds ratio; WLC, waiting-list control. Values are posterior median (95% CrI). | | |

**Supplementary Table 7. SUCRA by outcome: main vs age-excluded sensitivity analysis.**

| **Outcome** | **Treatment** | **Main SUCRA** | **Age-excluded SUCRA** | **SUCRA change** |
| --- | --- | --- | --- | --- |
| SAD severity | ICT | 0.955 |  |  |
| SAD severity | SET | 0.923 |  |  |
| SAD severity | ICBT | 0.712 | 0.842 | 0.130 |
| SAD severity | ET | 0.500 | 0.656 | 0.156 |
| SAD severity | VR | 0.487 | 0.642 | 0.155 |
| SAD severity | ICALM | 0.465 |  |  |
| SAD severity | VRAG | 0.460 | 0.586 | 0.126 |
| SAD severity | ISUPPORT | 0.451 |  |  |
| SAD severity | CBT | 0.442 | 0.252 | -0.190 |
| SAD severity | IPDT | 0.410 | 0.533 | 0.123 |
| SAD severity | CBM | 0.370 |  |  |
| SAD severity | IIPT | 0.370 | 0.399 | 0.029 |
| SAD severity | BCF | 0.323 | 0.411 | 0.088 |
| SAD severity | WLC | 0.131 | 0.179 | 0.048 |
| Depression | ICT | 0.966 |  |  |
| Depression | ICBT | 0.767 | 0.852 | 0.085 |
| Depression | ET | 0.663 | 0.816 | 0.153 |
| Depression | IPDT | 0.633 | 0.794 | 0.161 |
| Depression | ISUPPORT | 0.588 |  |  |
| Depression | BCF | 0.433 | 0.507 | 0.074 |
| Depression | VR | 0.407 | 0.492 | 0.085 |
| Depression | WLC | 0.315 | 0.364 | 0.049 |
| Depression | CBT | 0.220 | 0.173 | -0.047 |
| Depression | IIPT | 0.008 | 0.001 | -0.007 |
| Quality of life | VR | 0.986 | 0.981 | -0.005 |
| Quality of life | ISUPPORT | 0.677 |  |  |
| Quality of life | ET | 0.521 | 0.537 | 0.016 |
| Quality of life | ICBT | 0.498 | 0.579 | 0.081 |
| Quality of life | IIPT | 0.193 | 0.251 | 0.058 |
| Quality of life | WLC | 0.125 | 0.152 | 0.027 |
| Response (efficacy) | ICBT | 0.791 | 0.792 | 0.001 |
| Response (efficacy) | SICBT | 0.771 | 0.755 | -0.016 |
| Response (efficacy) | IPDT | 0.757 | 0.805 | 0.048 |
| Response (efficacy) | CBT | 0.597 | 0.605 | 0.008 |
| Response (efficacy) | SET | 0.576 |  |  |
| Response (efficacy) | VR | 0.534 | 0.253 | -0.281 |
| Response (efficacy) | CBM | 0.329 | 0.300 | -0.029 |
| Response (efficacy) | WLC | 0.260 | 0.245 | -0.015 |
| Response (efficacy) | ET | 0.235 | 0.645 | 0.410 |
| Response (efficacy) | IIPT | 0.151 | 0.100 | -0.051 |

**Supplementary Table 8. Convergence: maximum Gelman–Rubin PSRF (values near 1.00 indicate convergence).**

| Outcome | Main (max PSRF) | Age-excluded (max PSRF) |
| --- | --- | --- |
| SAD severity | 1.001 | 1.000 |
| Depression | 1.001 | 1.002 |
| Quality of life | 1.021 | 1.004 |
| Response (efficacy) | 1.001 | 1.006 |
