# Supplementary figures and images for: Efficacy of digital interventions in social anxiety disorder: a systematic review and Bayesian network meta-analysis
Source: Front Psychiatry. 2026 Jul 10;17:1883150. doi: 10.3389/fpsyt.2026.1883150 (PMC13397225; doi:10.3389/fpsyt.2026.1883150)

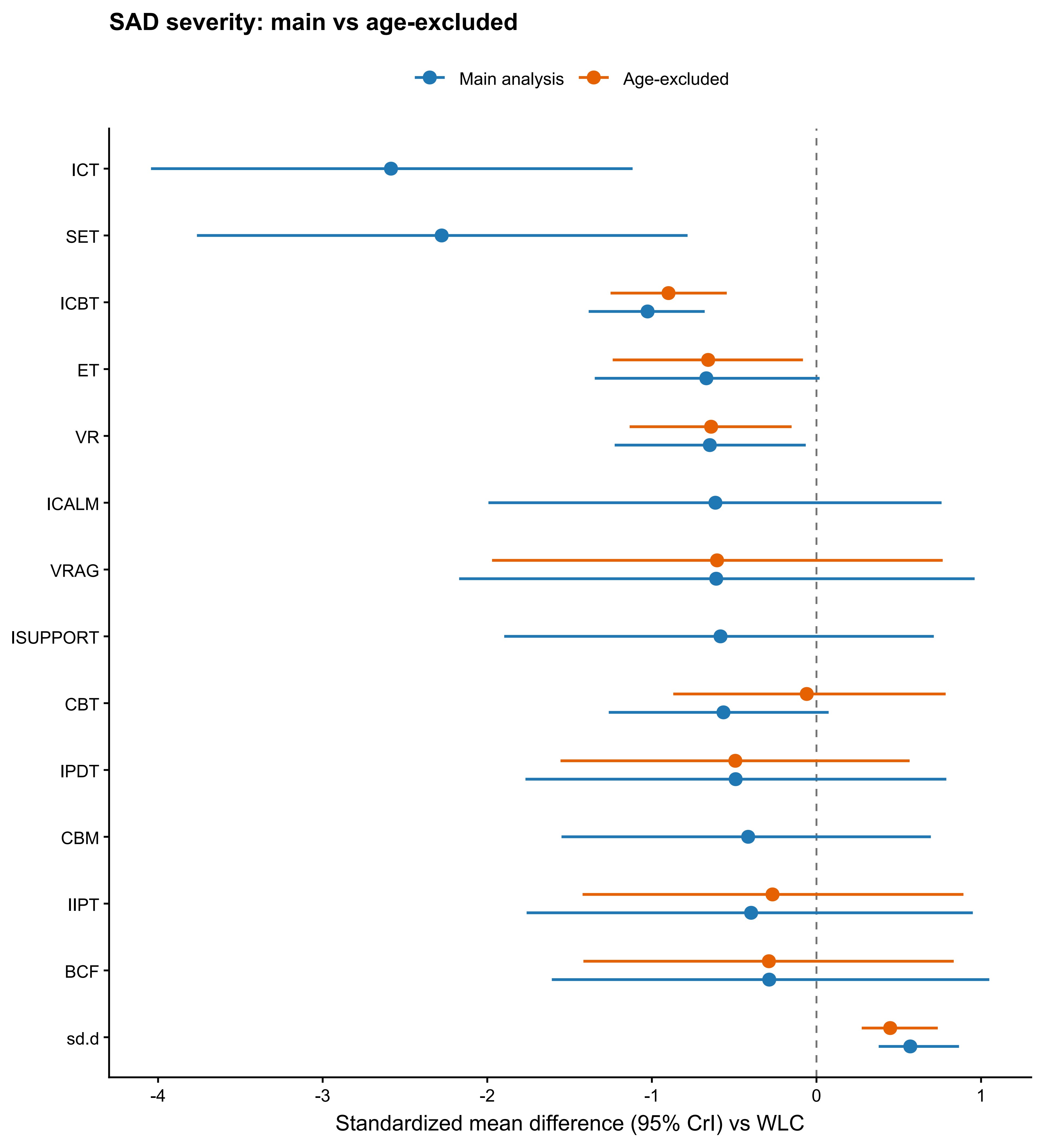

Supplement: Supplementary Figure 1 — Hot spot mapping. Two studies simultaneously included researchers from Germany, Switzerland, and Austria, and a total of 349 individuals were not included in the heat map. Map lines delineate study areas and do not necessarily depict accepted national boundaries. [file DataSheet1.zip › Supplementary Figure 10.png]

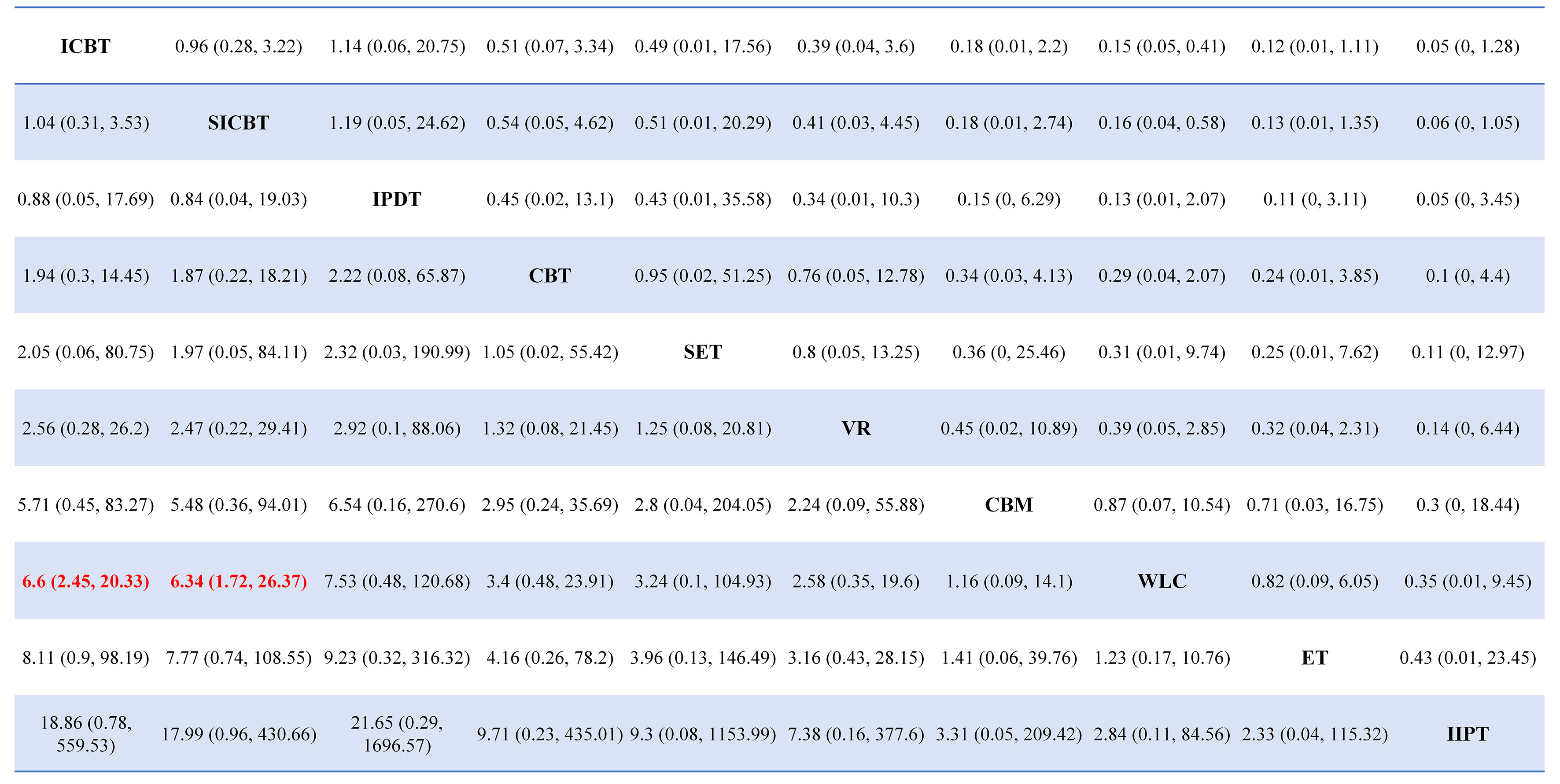

Supplement: Supplementary Figure 1 — Hot spot mapping. Two studies simultaneously included researchers from Germany, Switzerland, and Austria, and a total of 349 individuals were not included in the heat map. Map lines delineate study areas and do not necessarily depict accepted national boundaries. [file DataSheet1.zip › Supplementary Figure 5.jpg]

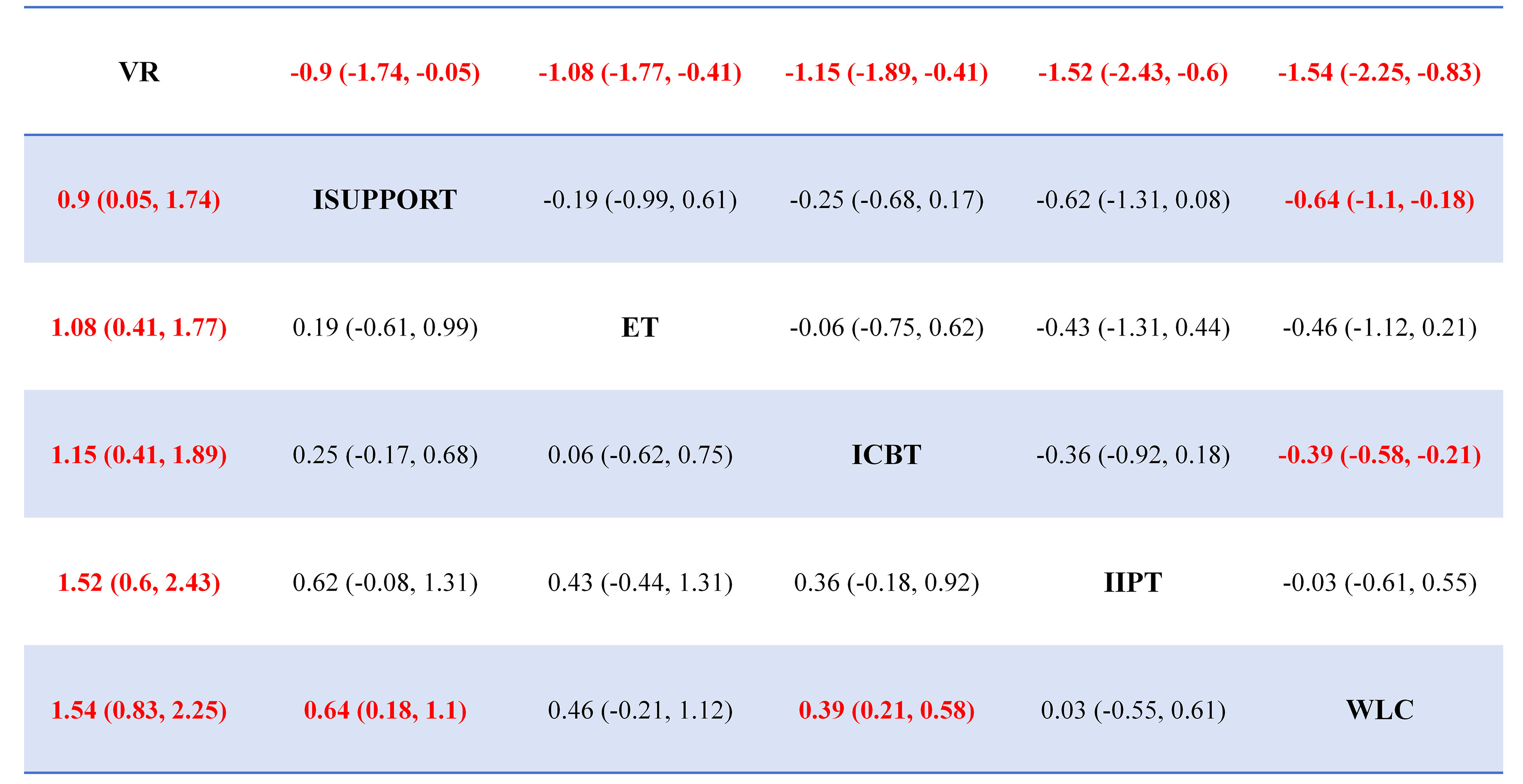

Supplement: Supplementary Figure 1 — Hot spot mapping. Two studies simultaneously included researchers from Germany, Switzerland, and Austria, and a total of 349 individuals were not included in the heat map. Map lines delineate study areas and do not necessarily depict accepted national boundaries. [file DataSheet1.zip › Supplementary Figure 4.jpg]

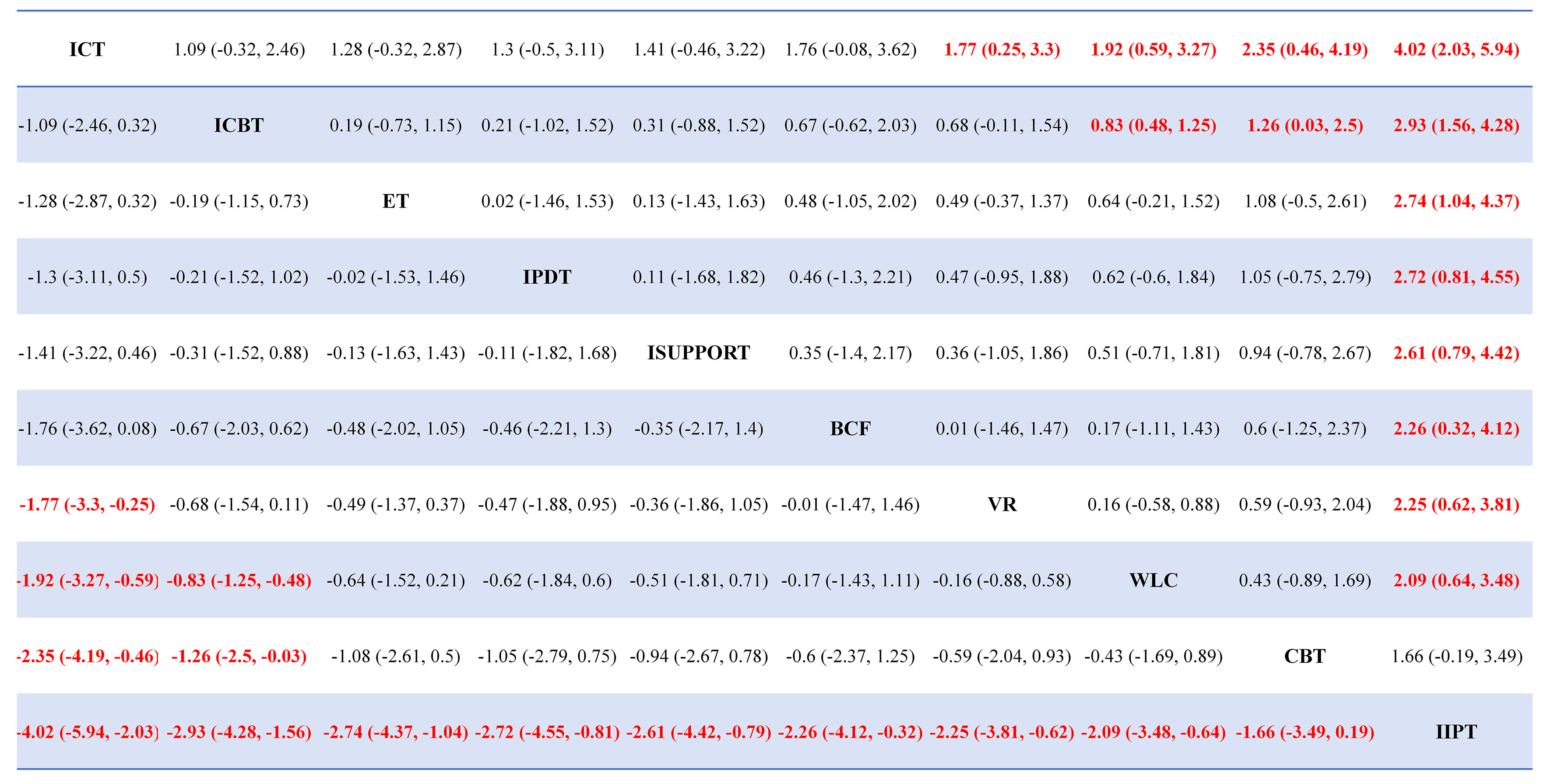

Supplement: Supplementary Figure 1 — Hot spot mapping. Two studies simultaneously included researchers from Germany, Switzerland, and Austria, and a total of 349 individuals were not included in the heat map. Map lines delineate study areas and do not necessarily depict accepted national boundaries. [file DataSheet1.zip › Supplementary Figure 3.jpg]

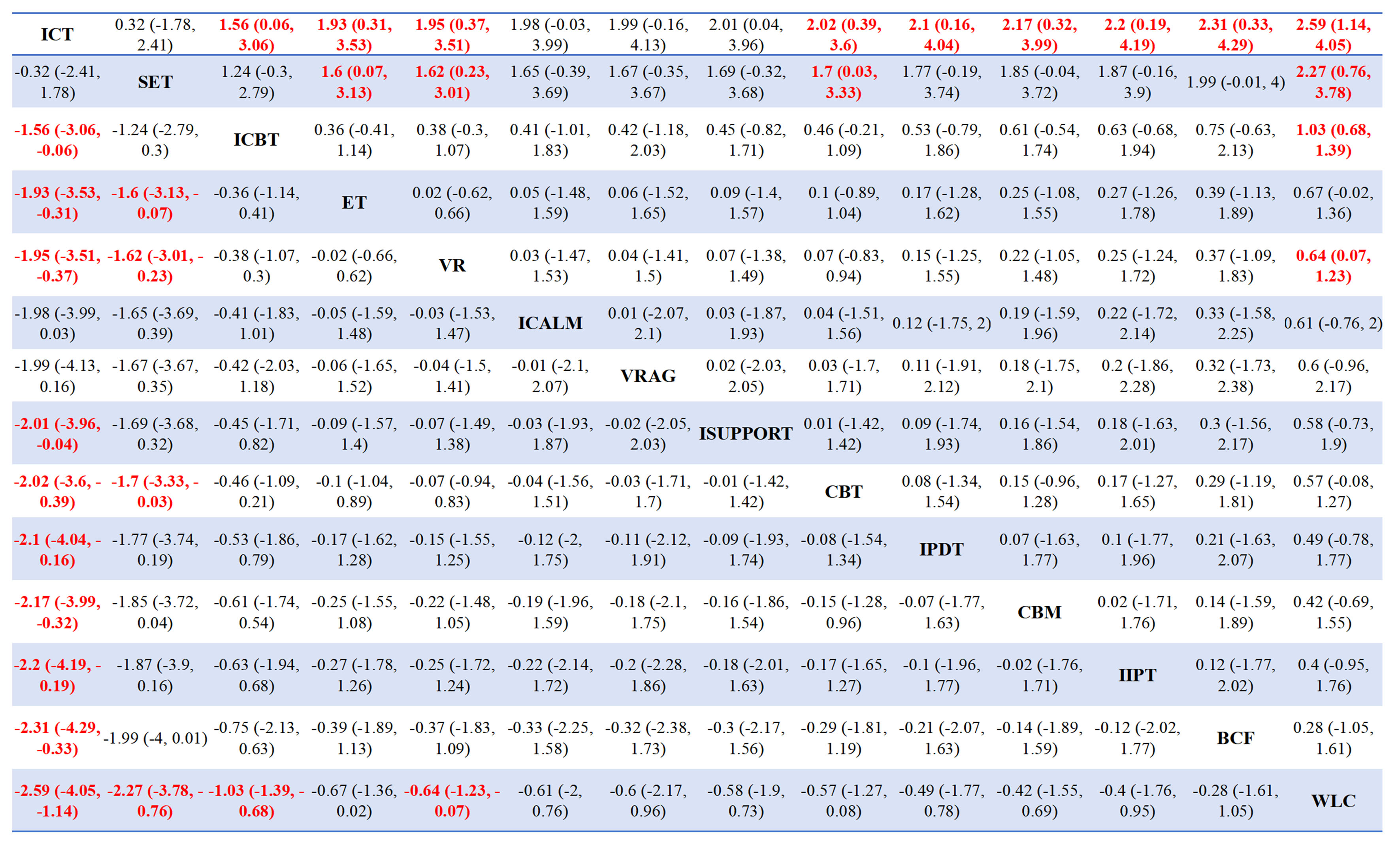

Supplement: Supplementary Figure 1 — Hot spot mapping. Two studies simultaneously included researchers from Germany, Switzerland, and Austria, and a total of 349 individuals were not included in the heat map. Map lines delineate study areas and do not necessarily depict accepted national boundaries. [file DataSheet1.zip › Supplementary Figure 2.jpg]

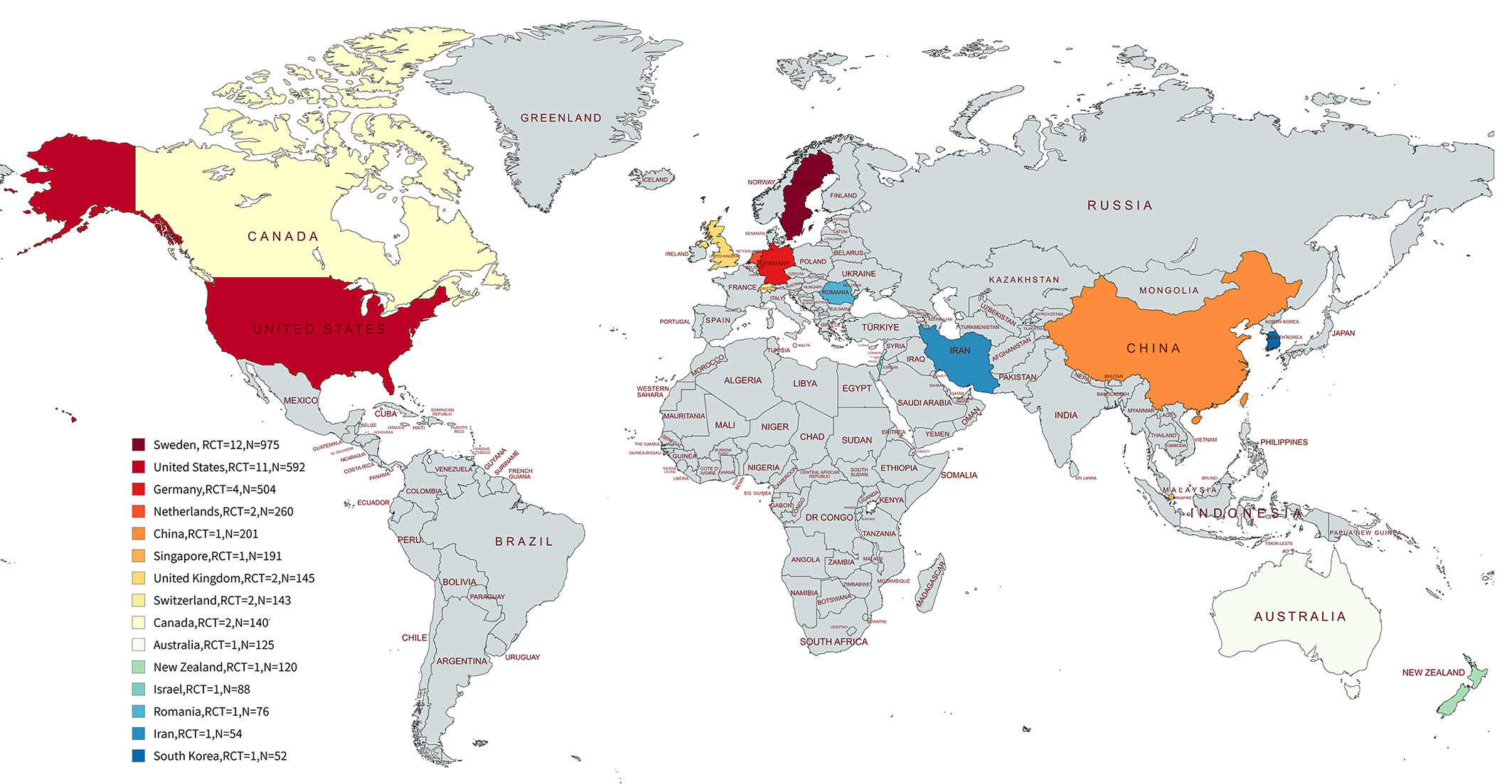

Supplement: Supplementary Figure 1 — Hot spot mapping. Two studies simultaneously included researchers from Germany, Switzerland, and Austria, and a total of 349 individuals were not included in the heat map. Map lines delineate study areas and do not necessarily depict accepted national boundaries. [file DataSheet1.zip › Supplementary Figure 1.jpg]

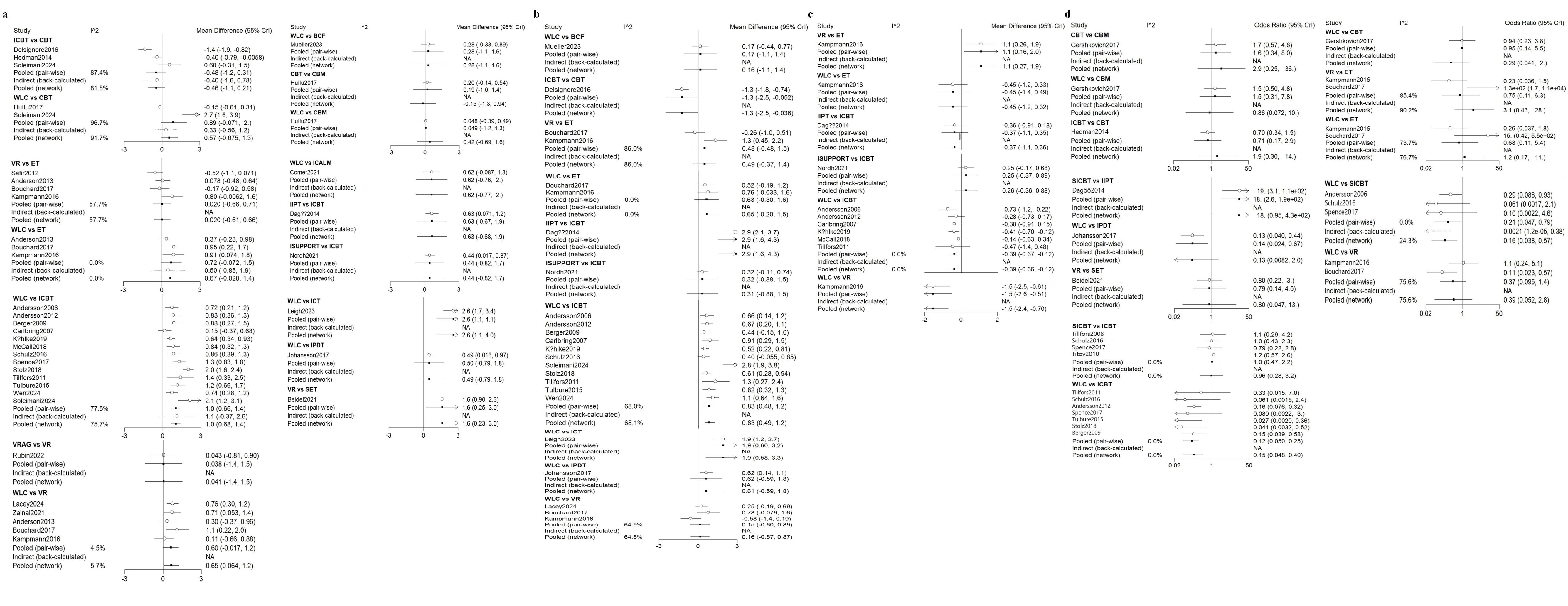

Supplement: Supplementary Figure 1 — Hot spot mapping. Two studies simultaneously included researchers from Germany, Switzerland, and Austria, and a total of 349 individuals were not included in the heat map. Map lines delineate study areas and do not necessarily depict accepted national boundaries. [file DataSheet1.zip › Supplementary Figure 8.jpg]

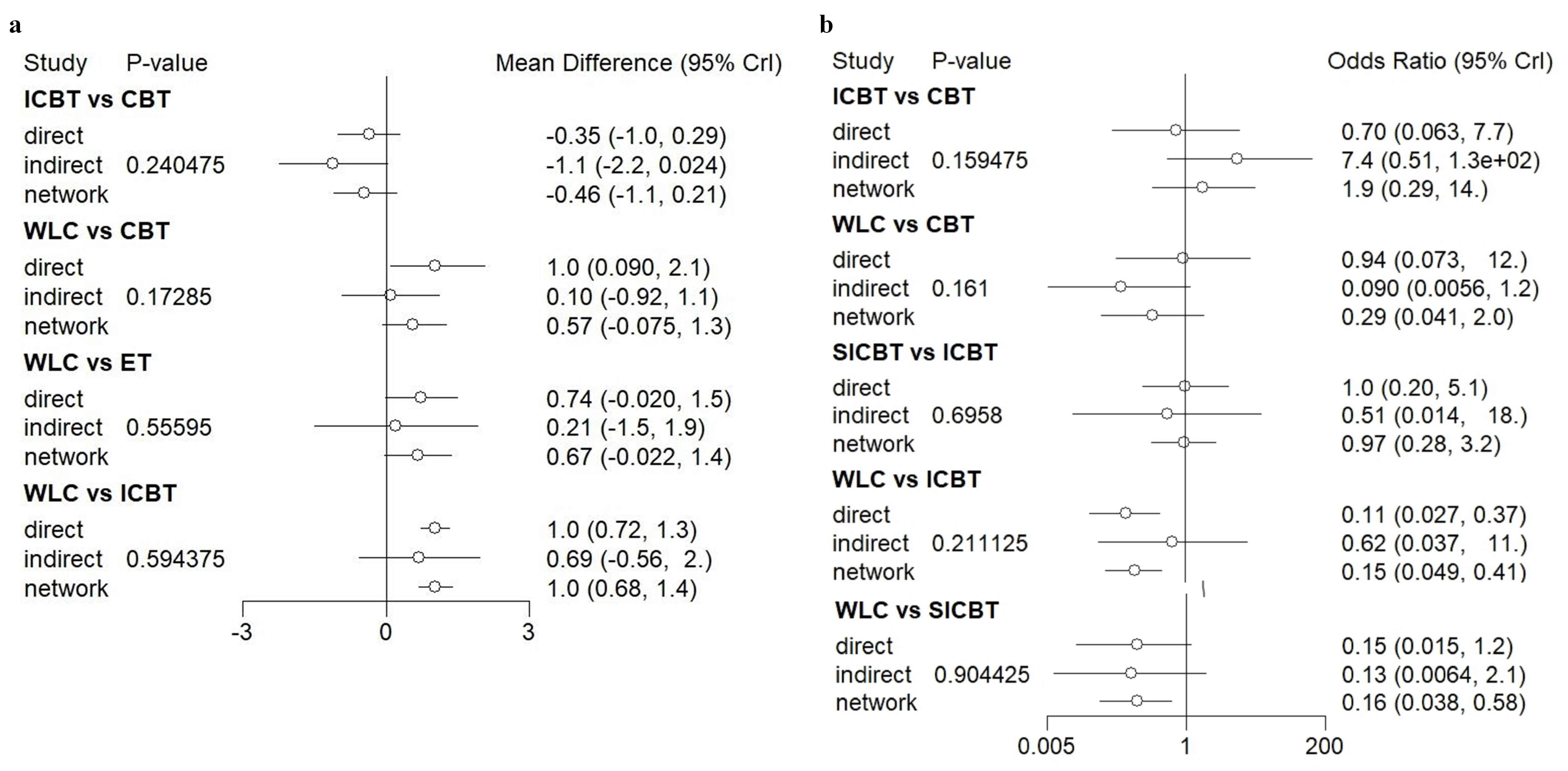

Supplement: Supplementary Figure 1 — Hot spot mapping. Two studies simultaneously included researchers from Germany, Switzerland, and Austria, and a total of 349 individuals were not included in the heat map. Map lines delineate study areas and do not necessarily depict accepted national boundaries. [file DataSheet1.zip › Supplementary Figure 9.jpg]

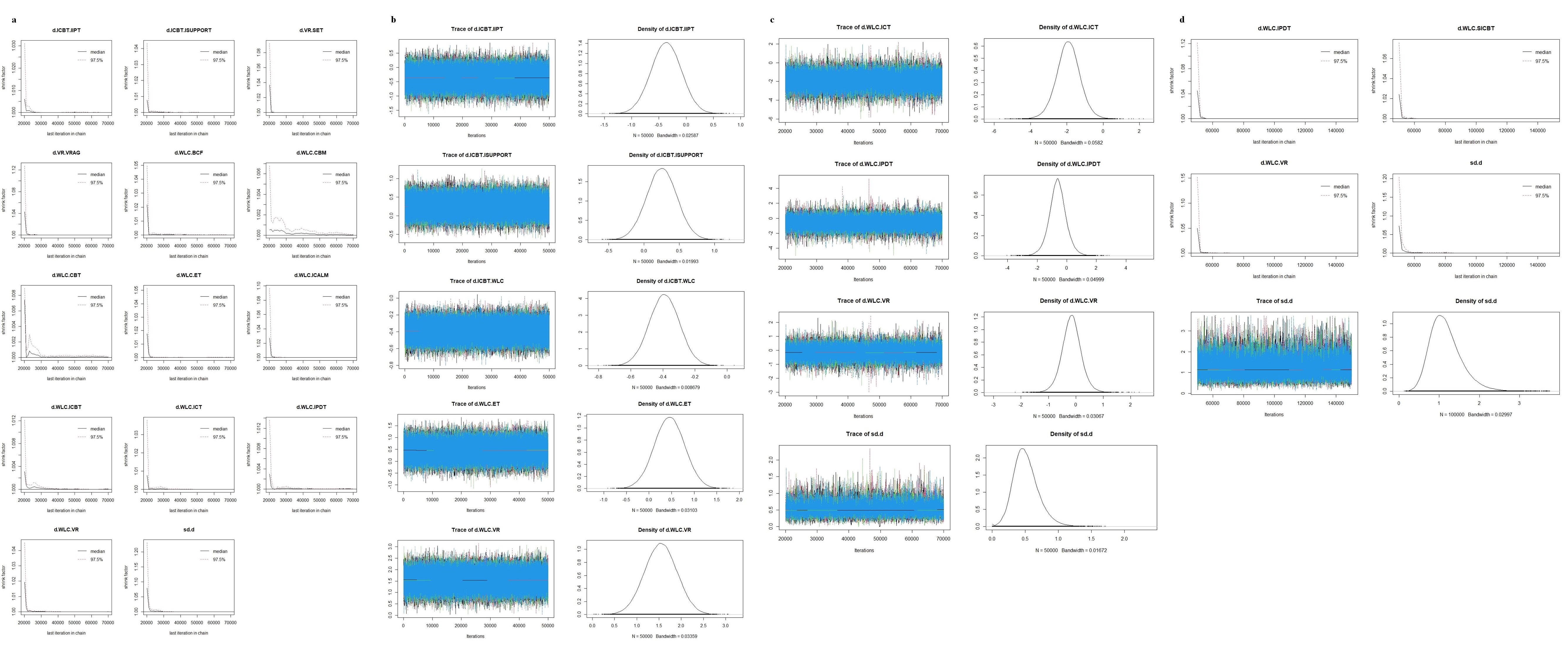

Supplement: Supplementary Figure 1 — Hot spot mapping. Two studies simultaneously included researchers from Germany, Switzerland, and Austria, and a total of 349 individuals were not included in the heat map. Map lines delineate study areas and do not necessarily depict accepted national boundaries. [file DataSheet1.zip › Supplementary Figure 7.jpg]

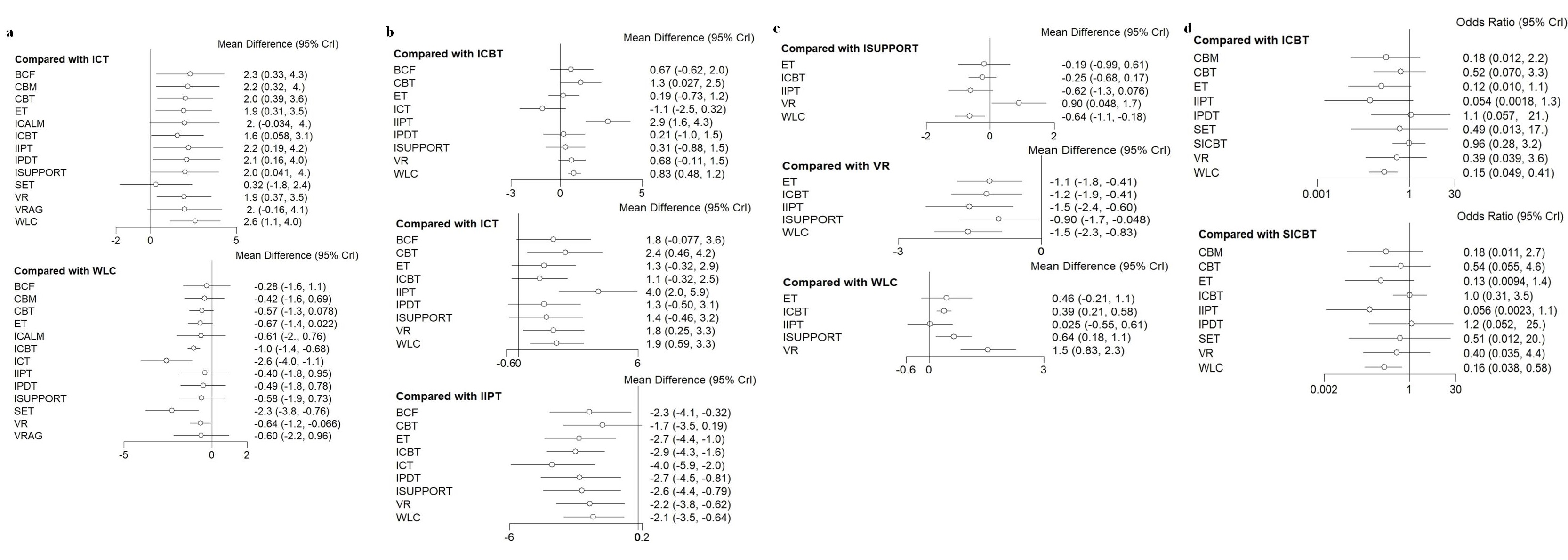

Supplement: Supplementary Figure 1 — Hot spot mapping. Two studies simultaneously included researchers from Germany, Switzerland, and Austria, and a total of 349 individuals were not included in the heat map. Map lines delineate study areas and do not necessarily depict accepted national boundaries. [file DataSheet1.zip › Supplementary Figure 6.jpg]
